# Supplementary material for: The Molecular Basis for the Endocytosis of Small R-SNAREs by the Clathrin Adaptor CALM
Source: Cell. 2011 Nov 23;147(5):1118–31. doi: 10.1016/j.cell.2011.10.038 (PMC3267021; doi:10.1016/j.cell.2011.10.038)
Supplement: Table S1. Data Collection and Refinement Statistics, Related to Figures 4 and 5 [file mmc1.pdf]

Table S1. Data Collection and Refinement Statistics

Numbers in parentheses refer to the appropriate outer shell.

**Data Collection**	**$CALM_{ANTH(1-289)}$**	**$CALM_{ANTH(1-264)}$**	**$CALM_{ANTH(1-264)}$:$VAMP8_{(11-41)}$**
Beamline	Diamond I04	Diamond I03	Diamond I03
Detector	ADSC Q315 CCD	ADSC Q315 CCD	ADSC Q315 CCD
Wavelength (Å)	0.9763	0.9763	0.9763
Resolution limits (Å)	73.5–1.8 (1.85–1.80)	60.5–1.7 (1.74–1.70)	91.1–2.0 (2.08–2.03)
Space group	$P2_12_12_1$	$C2$	$C2$
Unit cell dimensions (Å, °)	a = 73.5<br>b = 78.3<br>c = 120.2	a = 95.9<br>b = 121.1<br>c = 62.4<br>ß = 110.7	a = 161.1<br>b = 100.3<br>c = 104.0<br>ß = 118.9
Unique reflections	64,198 (4,652)	72,568 (5,397)	93,133 (6,910)
Redundancy	14.1 (14.5)	4.3 (4.3)	3.7 (3.7)
Completeness (%)	98.9 (98.1)	99.4 (99.9)	99.7 (99.8)
$<I/\sigma(I)>$	14.4 (3.1)	9.8 (2.1)	10.0 (1.9)
R_{merge}[a]	0.100 (0.862)	0.071 (0.484)	0.079 (0.731)
R_{meas}[b]	0.104 (0.893)	0.081 (0.553)	0.093 (0.855)
R_{pim}[c]	0.028 (0.231)	0.038 (0.264)	0.048 (0.440)
**Refinement**			
Resolution limits (Å)	40.0–1.8 (1.83–1.80)	35.5–1.7 (1.72–1.70)	45.6–2.0 (2.05–2.03)
Reflections in working set	64,141 (2,629)	72,462 (2,670)	93,106 (2,933)
Reflections in test set	3,237 (115)	3,652 (138)	4,667 (162)
R_{work} (%)[d]	0.182 (0.301)	0.170 (0.288)	0.182 (0.294)
R_{free} (%)[e]	0.216 (0.347)	0.191 (0.300)	0.199 (0.365)
Number of molecules/complexes per asymmetric unit	2	2	3
Number of atoms (protein/water/other[f])	4,417/415/0	4,225/444/0	6,844/473/8
Residues in Ramachandran favored region (%)	98.7	98.3	99.2
Ramachandran outliers (%)	0.0	0.0	0.0
r.m.s.d bond lengths (Å)[g]	0.009	0.010	0.008
r.m.s.d bond angles (°)[g]	1.077	1.110	0.911
Average B factors (Å^2) (protein/solvent)	35.1/42.0	31.1/40.68	41.2/46.8

[a]$R_{merge} = \Sigma_{hkl} \Sigma_i |I(hkl;i) - \langle I(hkl)\rangle| / \Sigma_{hkl} \Sigma_i I(hkl;i)$, where $I(hkl;i)$ is the intensity of an individual measurement of a reflection and $\langle I(hkl)\rangle$ is the average intensity of that reflection.

[b]R_{meas} is the redundancy-independent merging R factor (Diederichs and Karplus, 1997).

[c]R_{pim} is the precision-indicating merging R factor (Weiss et al., 1998).

[d]$R_{work} = \Sigma_{hkl}||F_{obs}| - |F_{calc}||/\Sigma_{hkl} |F_{obs}|$, where $|F_{obs}|$ and $|F_{calc}|$ are the observed and calculated structure factor amplitudes, respectively.

[e]R_{free} equals R of the cross-validation (test) set, 5% of the data removed prior to refinement.

[f]Other indicates PO_4^{3-} ions.

[g]r.m.s.d. is root mean square deviation from ideal geometry.
