# Supplementary material for: The Molecular Basis for the Endocytosis of Small R-SNAREs by the Clathrin Adaptor CALM
Source: Cell. 2011 Nov 23;147(5):1118–31. doi: 10.1016/j.cell.2011.10.038 (PMC3267021; doi:10.1016/j.cell.2011.10.038)
Supplement: Document S1. Article plus Supplemental Information [file mmc2.pdf]

Cell

The Molecular Basis for the Endocytosis of Small R-SNAREs by the Clathrin Adaptor CALM

Sharon E. Miller,[1,3] Daniela A. Sahlender,[1,3] Stephen C. Graham,[1] Stefan Höning,[2] Margaret S. Robinson,[1] Andrew A. Peden,[1,*] and David J. Owen[1,*]
[1]Cambridge Institute for Medical Research and Department of Clinical Biochemistry, University of Cambridge, Addenbrooke's Hospital, Hills Road, Cambridge CB2 0XY, UK
[2]Institute of Biochemistry I and Center for Molecular Medicine Cologne, University of Cologne, Joseph-Stelzmann-Str. 52, 50931 Cologne, Germany
[3]These authors contributed equally to this work
*Correspondence: aap20@cam.ac.uk (A.A.P.), djo30@cam.ac.uk (D.J.O.)
DOI 10.1016/j.cell.2011.10.038

SUMMARY

SNAREs provide a large part of the specificity and energy needed for membrane fusion and, to do so, must be localized to their correct membranes. Here, we show that the R-SNAREs VAMP8, VAMP3, and VAMP2, which cycle between the plasma membrane and endosomes, bind directly to the ubiquitously expressed, PtdIns4,5P_2-binding, endocytic clathrin adaptor CALM/PICALM. X-ray crystallography shows that the N-terminal halves of their SNARE motifs bind the $CALM_{ANTH}$ domain as helices in a manner that mimics SNARE complex formation. Mutation of residues in the CALM:SNARE interface inhibits binding in vitro and prevents R-SNARE endocytosis in vivo. Thus, CALM:R-SNARE interactions ensure that R-SNAREs, required for the fusion of endocytic clathrin-coated vesicles with endosomes and also for subsequent postendosomal trafficking, are sorted into endocytic vesicles. CALM's role in directing the endocytosis of small R-SNAREs may provide insight into the association of CALM/PICALM mutations with growth retardation, cognitive defects, and Alzheimer's disease.

INTRODUCTION

SNAREs (Soluble NSF Attachment Protein REceptors) are small membrane-anchored proteins that lie at the heart of the vesicle:organelle and organelle:organelle membrane fusion machinery, providing much of the energy and specificity required for membrane fusion (Hong, 2005; Jahn and Scheller, 2006; Sutton et al., 1998). As with all membrane proteins, SNAREs must be positioned in their appropriate cellular location in order to function correctly. In recent years, it has become apparent that the cell possesses mechanisms for transporting SNAREs between its various membranes alongside standard (non-SNARE) cargo. Here, we investigate the molecular mechanism by which the SNAREs VAMP8, VAMP3, and VAMP2 are internalized from the plasma membrane.

There are at least 38 SNAREs in mammalian cells (Bock et al., 2001; Hong, 2005; Kloepper et al., 2007). Most contain a single conserved helical SNARE motif of 60–70 residues, although SNAP23, SNAP25, and SNAP29 contain two (Jahn and Scheller, 2006). N-terminal to their SNARE motifs, most SNAREs have a folded region that varies in length from 100–150 residues and is usually either a three helical H_{abc} domain or a longin domain (reviewed in Hong, 2005). SNARE complexes are formed when four SNARE motifs come together as a tetrameric coiled-coil (Sutton et al., 1998). Three of these SNARE motifs are associated with one membrane and derive from the so-called Q-SNAREs, while the other SNARE motif is provided by an R-SNARE that resides in the membrane that will fuse with the first membrane (Fasshauer et al., 1998). It is this relative orientation of the (Q-) and (R-) SNAREs that draws the two membranes close enough to drive their fusion. The specificity of vesicle:organelle and organelle:organelle fusion arising from the limited combinations of SNAREs that can form complexes can only come about if the localization of SNAREs is itself controlled. For instance, SNAREs must be transported to a given organelle membrane so that they can subsequently be sorted into transport vesicles and tubules leaving that membrane since this enables these transport vesicles/tubules to fuse, ultimately, with their desired target membrane, into which the correct cognate SNAREs must have already been placed.

The active sorting of SNAREs into transport vesicles/tubules is achieved primarily by direct interaction with components of the vesicle/tubule's protein coat, although transmembrane helix length may also play a role (Sharpe et al., 2010). Initial mechanistic descriptions of active SNARE sorting came from studies on COPII coated vesicles, which mediate ER to Golgi transport (Mancias and Goldberg, 2007; Mossessova et al., 2003). In post-Golgi trafficking, the sorting of Vti1b by EpsinR (Miller et al., 2007) and of VAMP7 by Hrb (Pryor et al., 2008) and AP3 (Martinez-Arca et al., 2003) are mediated by the direct interactions of the folded N-terminal domains of the SNAREs with the

respective coated vesicle adaptors. Since the molecular mechanisms by which these latter recognition events occur are distinct from those by which conventional short, linear motif (YxxΦ, ExxxLL, FxNPxY) containing cargo are recognized (Bonifacino and Traub, 2003), the two systems are noncompetitive and so can act in parallel to ensure that both SNAREs and cargo are incorporated into transport vesicles.

VAMP8 and VAMP3 cycle between the cell's limiting membrane and early endosomes/recycling endosomes and thus mediate the fusion of vesicles with both compartments, whereas VAMP2 drives the fusion of fast-recycling synaptic vesicles with the plasma membrane (Antonin et al., 2000; Grote et al., 1995; McMahon et al., 1993). None of these three SNAREs have a conventional cargo motif (such as the ExxxLL motif found on VAMP4 [Peden et al., 2001]), nor do they possess folded N-terminal domains. However, preceeding their SNARE motifs there are short regions of 10–30 residues that are predicted to be unstructured (Ellena et al., 2009; Fiebig et al., 1999; Hazzard et al., 1999). The question thus arises: how are these SNAREs sorted into endocytic clathrin-coated vesicles? Based on the observations that both Vti1b and VAMP7 can be transported as part of a cis-SNARE complex (Miller et al., 2007; Pryor et al., 2008), one possibility was that VAMPs 8, 3, and 2 could be internalized as a complex with their acceptor (Q-) SNAREs (a syntaxin and a SNAP) (Gordon et al., 2009). However, endocytic and synaptic vesicles contain a large excess of these small R-SNAREs over their acceptor (Q-) SNAREs (Takamori et al., 2006) and in addition, the steady state localization of VAMPs 8, 3, and 2 is endosomal, whereas that of syntaxins1-4 and SNAPs is at the plasma membrane. Together, these observations suggest that trafficking of small R-SNARE VAMPs as part of a complex is unlikely.

In this study, we provide structural, biochemical and in vivo data to show that the internalization of VAMP8, and consequently that of the highly related small R-SNAREs, VAMP3 and VAMP2, is mediated by specific, direct interactions between the N-terminal halves of their SNARE motifs and the ANTH (AP180 N-terminal Homology) domain of the endocytic clathrin adaptor CALM.

RESULTS

CALM Is Involved in the Endocytosis of VAMPs 2, 3, and 8

Recent in vivo studies (Harel et al., 2008) suggested that the ubiquitously expressed CALM and possibly its neuronal specific homolog AP180 (Dreyling et al., 1996; Lindner and Ungewickell, 1992; Yao et al., 2003), may play a role in the trafficking of VAMP2 in mammalian cells. This supported earlier work in *D. melanogaster* (Bao et al., 2005), *C. elegans* (Nonet et al., 1999), and yeast (Burston et al., 2009), suggesting that these organisms' single ANTH domain containing clathrin adaptor proteins were involved in the endocytosis of short endocytic R-SNARE VAMPs.

To confirm a role for CALM in the trafficking of VAMP2 and to show that CALM was similarly involved in endocytosis of the related small endocytic R-SNAREs VAMP3 and VAMP8 (Hong, 2005), we investigated the effect of CALM depletion on VAMP2, VAMP3, and VAMP8 endocytosis. The three SNAREs were tagged with a double HA epitope at their (lumenal) C-termini (termed VAMP2-HA, VAMP3-HA, and VAMP8-HA) (Gordon et al., 2009) and were stably expressed in HeLa cells at relatively low levels (Figure S1A available online). Depletion of CALM by siRNA caused all three SNAREs to accumulate on the cell surface (Figure 1A). To investigate the rate of endocytosis of the three HA-tagged VAMPs, we used a flow cytometry-based antibody uptake assay (as outlined in Figure S1B). In control cells, the three SNAREs were rapidly internalized, with 40%–60% of the prebound antibody already inside the cells after 5 min. However, when CALM was depleted, antibody uptake was negligible even after 30 min (Figure 1). These effects are considerably more dramatic than previously reported (Harel et al., 2008), presumably because we expressed the constructs at lower than endogenous levels so as not to saturate the intracellular sorting machinery. The inhibition of VAMP endocytosis was rescued by expression of Myc-tagged, full-length, siRNA-resistant CALM, indicating that the phenotype is specific and not an off-target effect (Figure 1B and Figure S1C). In agreement with previous studies (Harel et al., 2008; Huang et al., 2004), the endocytosis rates of standard cargoes such as EGF and TfR were not significantly altered by CALM depletion (Figure S1D).

VAMPs 2, 3, and 8 Bind Directly to $CALM_{ANTH}$

To investigate the possibility of a direct interaction between any of these small R-SNAREs and the ANTH domain of CALM (defined as residues 19–289 [Ford et al., 2001]), we carried out "GST pull-down" binding assays using recombinantly expressed proteins. As shown in Figure 2A the three SNAREs interacted directly with the $CALM_{ANTH}$ domain in a concentration-dependent manner. Isothermal titration calorimetry (ITC) was used to quantify the strength of binding. The interaction between GST-VAMP8 and $CALM_{ANTH}$ is the tightest with a K_D of $\sim$18 μM (Figure 2B), which is typical for a dynamic cargo/coat interaction (Owen et al., 2004; Pryor et al., 2008). Binding of the $CALM_{ANTH}$ domain to GST-VAMP3 and GST-VAMP2, although similar to one another ($\sim$46 μM and $\sim$43 μM respectively), were weaker than the binding to VAMP8 (Figure 2B). These differences in affinities are reflected in the relative rates of endocytosis of the three VAMPs (Figure 1B). No interaction could be detected between any of the three R-SNAREs and the related ENTH domain of the endocytic clathrin adaptor, epsin1.

The ANTH domains of CALM and neuronal specific AP180 share 82% sequence identity. However, in our hands, the ANTH domain of mammalian AP180 showed no significantly measurable binding to mammalian VAMPs 2, 3, or 8 by GST pull-downs or surface plasmon resonance (SPR) (Figure S2A and S2B), despite being correctly folded as indicated by circular dichroism (CD) (data not shown) and being competent to bind PtdIns(4,5)P_2 similarly to $CALM_{ANTH}$ by ITC (Figure S2C) and liposome-based SPR (data not shown). We have no simple explanation for this, but note that AP180 and CALM are not functionally redundant (Bushlin et al., 2008; Harel et al., 2008) and that the single ANTH domain containing clathrin adaptor in lower organisms is more like CALM than AP180 (Harel et al., 2008).

Taken together with the in vivo studies published by others and presented here, our biochemical data point to the direct

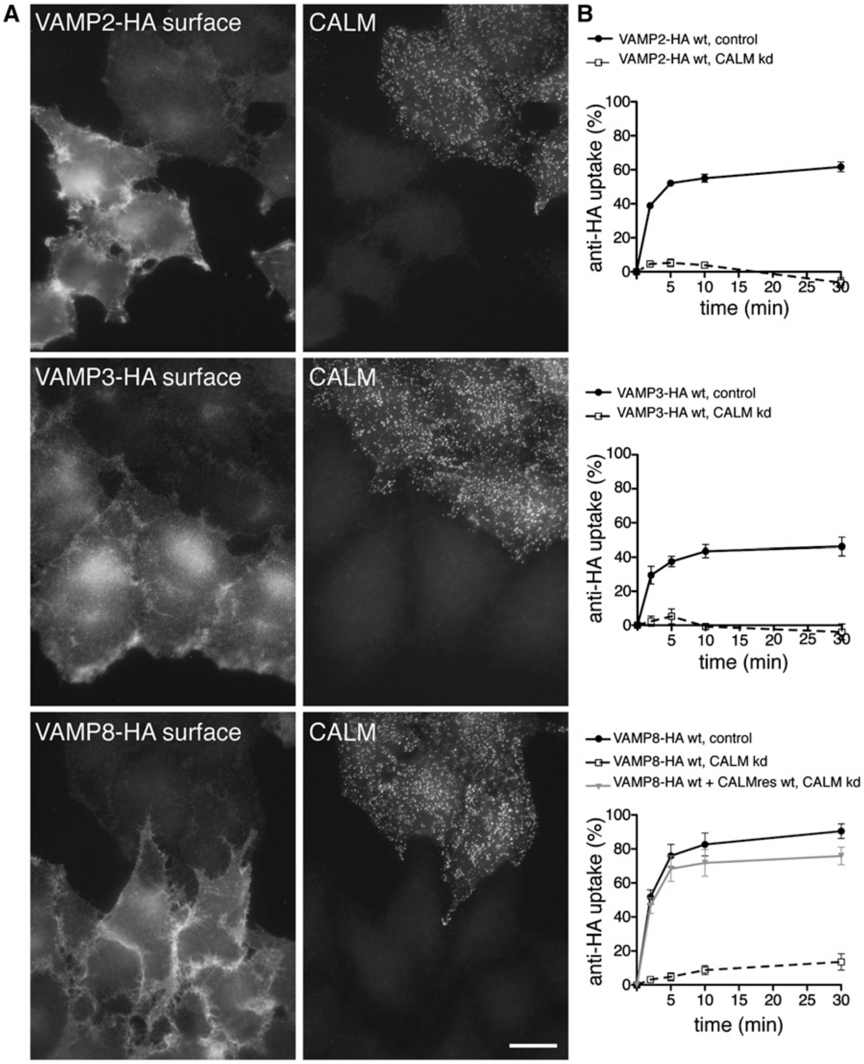

Figure 1. CALM Depletion Abolishes Endocytosis of HA-Tagged VAMPs

(A) Effect of CALM depletion on surface expression of HA-tagged VAMPs. Control and siRNA-treated cells expressing HA-tagged VAMP2, VAMP3, or VAMP8 were mixed together, fixed without permeabilization, and labeled with anti-HA, then permeabilized and labeled with anti-CALM. Knocking down CALM increases the surface expression of all three VAMPs. The scale bar represents 20 μm.

(B) Endocytosis of anti-HA in cells expressing HA-tagged VAMPs. Antibody was bound to the cells at 4°C, then the cells were warmed to 37°C for 2–30 min and antibody remaining at the cell surface was quantified by flow cytometry. Each point is derived from at least three separate experiments; the error bars show the SEM. Knocking down CALM effectively abolishes the uptake of all three VAMPs. The specificity of the knockdown phenotype was demonstrated by stably transfecting VAMP8-HA-expressing cells with siRNA-resistant myc-tagged CALM (CALMres wt) and knocking down endogenous CALM. Expression of the CALM construct almost completely rescues the knockdown phenotype.

See also Figure S1.

binding of the VAMPs 2, 3, and 8 by CALM being directly responsible for the endocytosis of these small R-SNAREs. Hence, we set out to investigate the molecular basis for this interaction using a combination of structure determination and in vitro and in vivo assays using wild-type and structure-directed mutant versions of both proteins. Because their sequence similarities (Figure 3A) and similar binding affinities indicated a conserved mechanism of interaction for the three SNARES, for technical reasons we characterized at the structural level only the interaction between $CALM_{ANTH}$ and the R-SNARE to which it bound most tightly, VAMP8.

The N-Terminal Half of the VAMP8 SNARE Motif Binds to $CALM_{ANTH}$

In order to delineate which portion of VAMP8 binds to $CALM_{ANTH}$, a number of deletion constructs were made (Figure 3B), an approach we deemed acceptable since several studies (Fasshauer et al., 1997; Fiebig et al., 1999; Hazzard et al., 1999) have shown small VAMPs to have no secondary structure in solution. GST pull-down experiments using truncated versions of VAMP8 (Figure 3C) revealed that the minimal region necessary for binding was residues 10-41 i.e., the N-terminal half of the SNARE motif. ITC showed that the binding of residues 10-41 of VAMP8 was almost identical (K_D ~20 μM) to that of wild-type VAMP8 (K_D ~18 μM) (Figure 3D), indicating that all the determinants for CALM binding are contained within residues 10-41. Significantly, the corresponding regions of mammalian VAMP2 and of the yeast endocytic VAMP Snc1p contains two key residues, Val43 and Met46 (VAMP2 numbering), which, when mutated to alanines, blocks their endocytosis (Grote et al., 1995; Lewis et al., 2000). In agreement with these data, we found that mutation of these residues to alanines in VAMP2-HA and likewise of the analogous residues in VAMP3-HA (Val30 and Met33) and VAMP8-HA (Lys24 and Met27) blocked their internalisation from the cell surface (Figures 3E and S3).

The VAMP8 SNARE Motif Binds in Place of the $CALM_{ANTH}$ Final Helix

Attempts were made to crystallize a complex of $CALM_{ANTH}$ with a peptide corresponding to residues 10–41 of VAMP8, both with the two components free in solution and with residues 10–41 of VAMP8 appended on the C terminus of $CALM_{ANTH}$. While these cocrystallizations did not yield the structure of a complex, they did yield a 1.8Å resolution structure (final R/R_{free} 0.182/0.216; see Table S1) of the unliganded ANTH domain with two molecules in the asymmetric unit. In both molecules the positions of

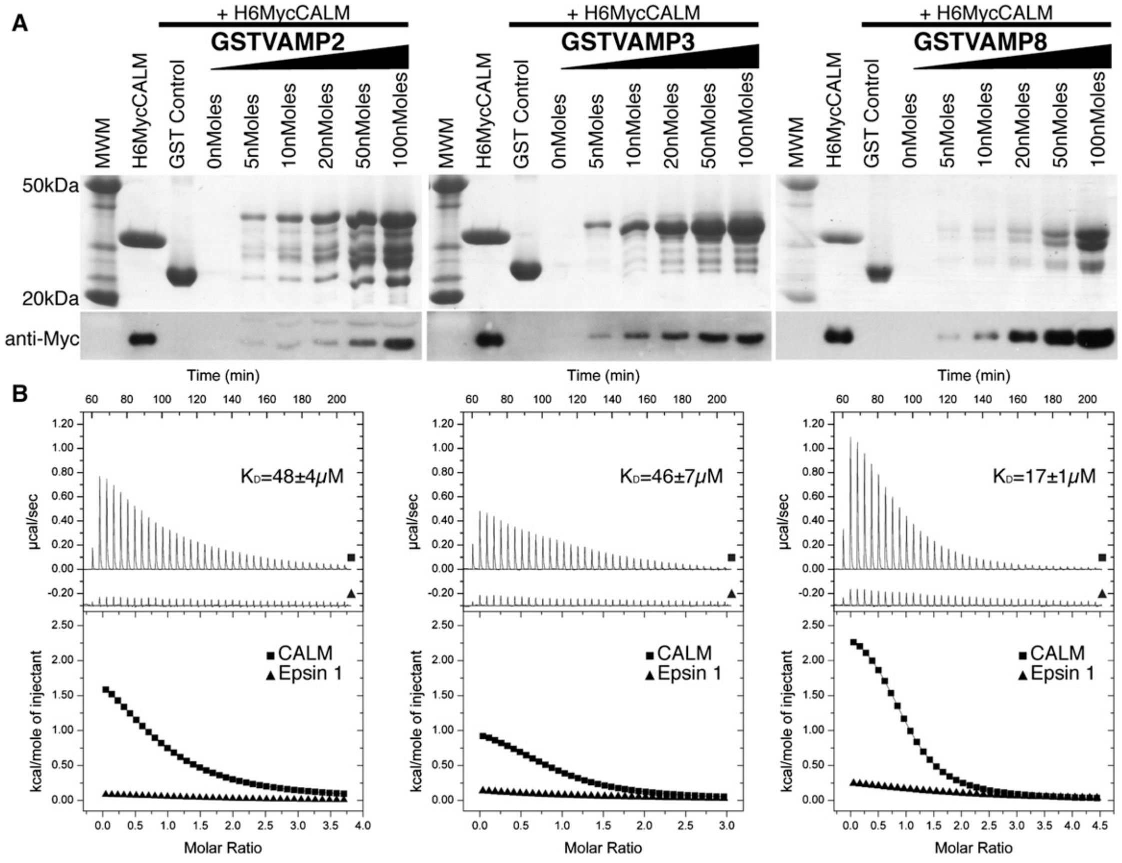

Figure 2. CALM Binds Directly to VAMP8, VAMP3, and VAMP2

(A) GST Pull downs using $His_6MycCALM_{ANTH}$ and the GST-fusion proteins indicated. Top: Coomassie blue stained gel. Lower: western blot probed with anti-myc. In this and all subsequent experiments, the lane adjacent to the Molecular weight markers (MWM) is loaded with $His_6MycCALM_{ANTH}$ only. The ANTH domain of CALM binds directly to VAMP2, 3, and 8 in a concentration dependent manner.

(B) ITC quantitating the binding of $CALM_{ANTH}$ to VAMP2, 3, and 8 (black squares). The Adaptor:SNARE interaction was tightest for VAMP8 (K_D17 ± 1µM) and weaker for both VAMP3 and VAMP2, (46 ± 7 µM and 48 ± 4 µM, respectively). $Epsin_{ENTH}$ showed no VAMP binding (black triangles) indicating the CALM:SNARE interaction is specific. Data for epsin1 is offset by −0.3 µcal/sec for clarity.

See also Figure S2.

the first ten helices are identical. The C-terminal 25 residues of the ANTH domain were poorly ordered in one molecule of the asymmetric unit, as in the published $CALM_{ANTH}$ structure determined from crystals of a different spacegroup (1HF8; Ford et al., 2001), while in the other molecule the C terminus was constrained by crystal packing and formed a four-turn helix (Figures 4 and S4A). The $CALM_{ANTH}$ structures presented here in conjunction with the previously published structure (Ford et al., 2001) suggested that residues 265–289 of CALM are not integral to the ANTH domain but are flexible and associate only weakly with the domain's core residues (19–264). Inspection of the $CALM_{ANTH}$ surface showed that the elongated patch on which the ordered short helix 11 and preceding ten residues of CALM sat was hydrophobic in nature and of the correct dimensions to bind an α helix and was thus a good candidate for the interaction site for the hydrophobic SNARE motif of a VAMP (Figure 4B). If this hydrophobic trough were indeed the VAMP binding site on $CALM_{ANTH}$, then deletion of helix 11 would be expected to increase VAMP8 binding. However, a truncated $CALM_{ANTH}$ construct missing helix 11 and the preceding ten residues, termed $CALM_{ANTH(1\text{-}264)}$, unexpectedly showed no binding to VAMP8 by GST pull-down experiments and ITC (Figures 4C and 4D) while still displaying normal binding to PtdIns4,5P_2 containing liposomes by SPR (data not shown). Multiangle light scattering (MALS) indicated that in solution $CALM_{ANTH(1\text{-}264)}$ is in fact a tight dimer (Figure S4B). This was confirmed by the determination of the structure of $CALM_{ANTH(1\text{-}264)}$ at 1.7Å resolution (final R/R_{free} 0.170/0.191; see Table S1). The dimeric structure explained the lack of VAMP8 binding since the dimer interface (burying 2100Å^2 of accessible surface area in total (Krissinel and Henrick, 2007)) was the proposed VAMP8 binding site (Figures 4E and 4F). As there were no other significant changes between the surfaces of $CALM_{ANTH}$ and $CALM_{ANTH(1\text{-}264)}$, these data strongly indicated that the hydrophobic trough in which helix 11 (when formed) sits was indeed the VAMP8 binding site on $CALM_{ANTH}$.

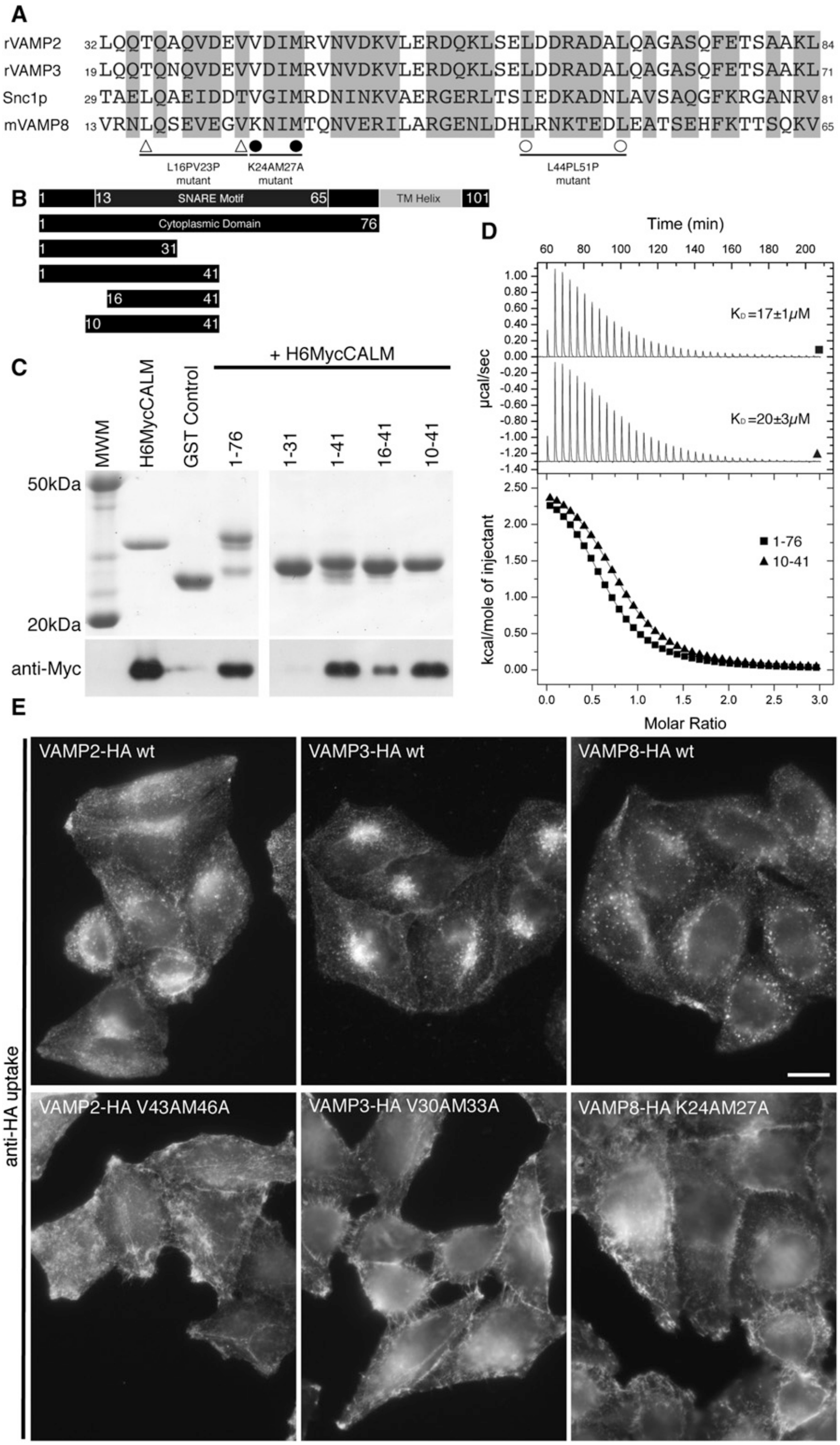

Figure 3. The N-Terminal Half of the Small R-SNARE VAMPs Binds to $CALM_{ANTH}$

(A) Sequence alignment of the SNARE motifs of VAMP2, VAMP3, Snc1p, and VAMP8. Conserved residues are boxed in gray. The position of mutated residues in VAMP8: L16 and V23 (open triangles), K24 and M27 (black circles), L44 and L51 (open circles) are indicated.

(B) Schematic representation of truncation mutants of VAMP8.

(C) GST pull-down experiments using $His_6MycCALM_{ANTH}$ and the GST-fusion proteins indicated. Top panel: Coomassie blue stained gel. Lower panel: western blot probed with anti-myc. The minimal fragment of VAMP8 that is able to bind to CALM with a similar affinity as the full cytoplasmic portion of VAMP8 (residues 1–76) comprises residues 10–41.

(D) ITC quantitating the binding of $CALM_{ANTH}$ to truncated VAMP8. $VAMP8_{10\text{-}41}$ (black triangles) exhibited an essentially identical binding affinity to $CALM_{ANTH}$ as $VAMP8_{1\text{-}76}$ (black squares) (K_Ds 17 ± 1 μM and 20 ± 3 μM respectively). Data for $VAMP8_{(10\text{-}41)}$ is offset by −1.3 μcal/s for clarity.

(E) Localization of anti-HA in cells expressing different wild-type or mutant HA-tagged VAMPs. The cells were allowed to endocytose the antibody for 40 min, then processed for immunofluorescence. The cells expressing wild-type VAMP2-HA, VAMP3-HA and VAMP8-HA have endocytosed the antibody, but the cells expressing the VAMP2-HAV43AM46A, VAMP3-HAV30AMetA, and VAMP8-HAK24AM27A mutants have retained the antibody on the plasma membrane. The scale bar represents 20 μm.

See also Figure S3.

Structure of a $CALM_{ANTH(1\text{-}264)}$: $VAMP8_{(11\text{-}41)}$ Chimeric Complex

In an attempt to obtain a structure of a complex between $CALM_{ANTH}$ and VAMP8, a construct was designed in which residues 11–41 of VAMP8 were fused through an artificial linker of six residues to residues 1–264 of $CALM_{ANTH}$. The resulting chimeric protein ($CALM_{ANTH(1\text{-}264)}$:$VAMP8_{(11\text{-}41)}$) crystallized and its 2.0 Å resolution structure (final R/R_{free} 0.183/0.199; see Table S1) showed unambiguously (Figures 5 and 6) that residues 15–38 of VAMP8 form a single α helix with residues between Phe16 and Arg37 contacting $CALM_{ANTH}$, consistent with the truncation data (Figure 3). The VAMP8 helix fitted into the spatially complementary groove of $CALM_{ANTH}$, lined by residues from helix 9 and helix 10, which was blocked by helix 11 in the unliganded $CALM_{ANTH}$ structures and by dimerization in $CALM_{ANTH(1\text{-}264)}$. There was a small movement of helices 9 and 10 with respect to each other that caused a slight widening of the groove as compared with the unliganded domain structure (Figure S5). No electron density was visible for 17 residues that connect the last ordered residue of CALM with the first of VAMP8. This would allow considerable flexibility in the positioning of the VAMP8 and indeed in the crystals where

it bound to $CALM_{ANTH}$ a VAMP8 SNARE motif could reach an adjacent ANTH domain to which it was not covalently linked.

Key residues in the interaction, 13 on VAMP8 and 16 on CALM, are spread throughout the interface between the two proteins (Figure 5). This shows that the binding is not a short, linear motif-mediated interaction that is typical of standard adaptor/cargo interactions. Eleven of the thirteen key interacting residues are conserved between VAMPs 8, 3, and 2 (Figure 3A), suggesting that they bind $CALM_{ANTH}$ in a similar manner. The residues in the $CALM_{ANTH}$:VAMP8 interface are highly conserved from human to fish. The interface is mainly hydrophobic in nature, burying a total of 1500$Å^2$ of accessible surface area (Krissinel and Henrick, 2007), and a full list of interacting residues is given in Figure 5D. The residues Lys24 and Met27 of VAMP8 play key roles in the interaction, explaining their initial identification as residues of importance for VAMP endocytosis (Grote et al., 1995) (Lewis et al., 2000). Mutation of these residues to alanines in VAMP8 abolished the interaction with $CALM_{ANTH}$ in vitro as shown by GST pull downs and quantified by ITC (Figures 6D and 6E), as did mutations of the analogous residues in VAMP2 and VAMP3 (data not shown). The single Met27Ala mutation when introduced into VAMP8 abolished the VAMP8:CALM interaction, whereas the Lys24Ala mutation on its own only weakened the interaction. The latter is to be expected since in VAMP2 and VAMP3, which also bind directly to $CALM_{ANTH}$ albeit more weakly, the equivalent residues are valines. When transferred to the in vivo situation, the non-CALM binding Lys24AlaMet27Ala mutant of VAMP8 accumulated on the cell surface and failed to be internalized (Figures 6H and 6I).

GST pull-down experiments demonstrated that mutation of hydrophobic residues in $CALM_{ANTH}$ that participate in VAMP8 binding, Leu219Ser, Phe240Ala, Met244Lys, Ile247Asp, and Leu251Ser, all abolished the interaction between the two proteins in vitro (Figures 6B and 6C) without affecting the fold (as judged by expression levels and circular dichroism, data not shown). Two of these, Leu219Ser and Met244Lys, whose lack of binding was confirmed by ITC (Figures 6B and 6C), were introduced into myc-tagged, siRNA-resistant CALM. When transfected into cells expressing VAMP8HA, depletion of endogenous CALM by siRNA caused the SNARE to be retained at the plasma membrane, because it could no longer be internalized (Figures 6F and 6G). This is in contrast to cells transfected with wild-type myc-tagged siRNA-resistant CALM, which retained the ability to internalize VAMP8HA upon endogenous CALM depletion (Figures 6F and 6G, 1B, and S1C). When taken together, the data presented in this study demonstrate that VAMP8 and the similar small R-SNAREs VAMP3 and VAMP2 are internalized from the plasma membrane by clathrin-mediated endocytosis due to a direct interaction with the clathrin adaptor CALM.

SNARE Complex Formation and Binding by CALM Are Mutually Exclusive Processes for VAMP8

Superposing the structure of the N-terminal half of the VAMP8 SNARE motif with the same portion of VAMP8 from the SNARE complex formed from VAMP8, Syntaxin7, Syntaxin8, and Vti1b (Antonin et al., 2002) demonstrates that, not only does VAMP8 adopt the same gently curving conformation in both complexes, but when the complex structures are overlayed via the VAMP8 molecules they contain, helices 9 and 10 of $CALM_{ANTH}$ superimpose on the SNARE motifs of Syntaxin8 and Syntaxin7, respectively (Figure 7A). Comparison of the two complex structures thus demonstrates that CALM binding and SNARE complex formation by VAMP8 must be mutually exclusive processes. This is confirmed by recombinant protein binding experiments, which show that GST-VAMP8 binds $CALM_{ANTH}$, but GST-VAMP8 complexed with SNAP23 and syntaxin3 (residues 195–253) does not (Figure 7B). It is important to note that the Lys24AlaMet27Ala, non-CALM binding mutant of VAMP8 cannot be internalized but, as we show in this work, is able to form SNARE complexes with its cognate plasma membrane SNARE partners. These observations show that, contrary to a previous proposal (Gordon et al., 2009), the ability of short R-SNAREs to form SNARE complexes and the ability to be endocytosed must in fact be independent of each other.

$CALM_{ANTH}$ Binds VAMP8 and PtdIns4,5P_2 Simultaneously

Since the binding sites on $CALM_{ANTH}$ for PtdIns4,5P_2 and a VAMP are on opposite ends of the ANTH domain, it is probable that both sites could bind simultaneously to their ligands. If this were indeed the case, avidity effects would result in greatly increased binding of CALM to a membrane containing both ligands when compared to the binding to membranes containing only one of the ligands, i.e., the K_D of the apparent binding to liposomes containing both ligands would be much lower than the average value of the individual K_Ds for the two ligands measured separately. To investigate whether or not simultaneous PtdIns4,5P_2 and VAMP binding does indeed occur, the binding of CALM to liposomes containing PC/PE/PtdIns4,5P_2, PC/PE+VAMP8 and PC/PE/PtdIns4,5P_2+VAMP8 was compared using liposome-based SPR. Figure 7C shows that indeed there is an almost order of magnitude decrease in K_D (i.e., increase in apparent affinity) over the average of the K_Ds for the individual ligands when both ligands are present in the same liposome membrane. Thus CALM must be able to bind simultaneously to PtdIns4,5P_2 and VAMP8.

Conformation of Membrane-Embedded VAMPs when Bound to CALM

Given that $CALM_{ANTH}$ can bind PtdIns4,5P_2 and a small R-SNARE VAMP simultaneously and based on the structural identification of the binding sites for a VAMP and PtdIns4,5P_2, the following deductions can be made. To allow simultaneous binding to a membrane-embedded VAMP and PtdIns4,5P_2 to occur, the amino-terminal half of the VAMP SNARE motif must be able to reach its binding site, which is at a distance of around 50Å from the membrane surface. Thus, the 38 residues between the end of the CALM binding sequence and the start of the transmembrane helix of a VAMP8 molecule must be able to stretch at least this far. The published NMR and CD studies and our own CD measurements (data not shown) indicate that in fact the entire cytoplasmic domain of short VAMPs has no secondary structure in solution (Fasshauer et al., 1997; Fiebig et al., 1999; Hazzard et al., 1999). In such an unstructured conformation,

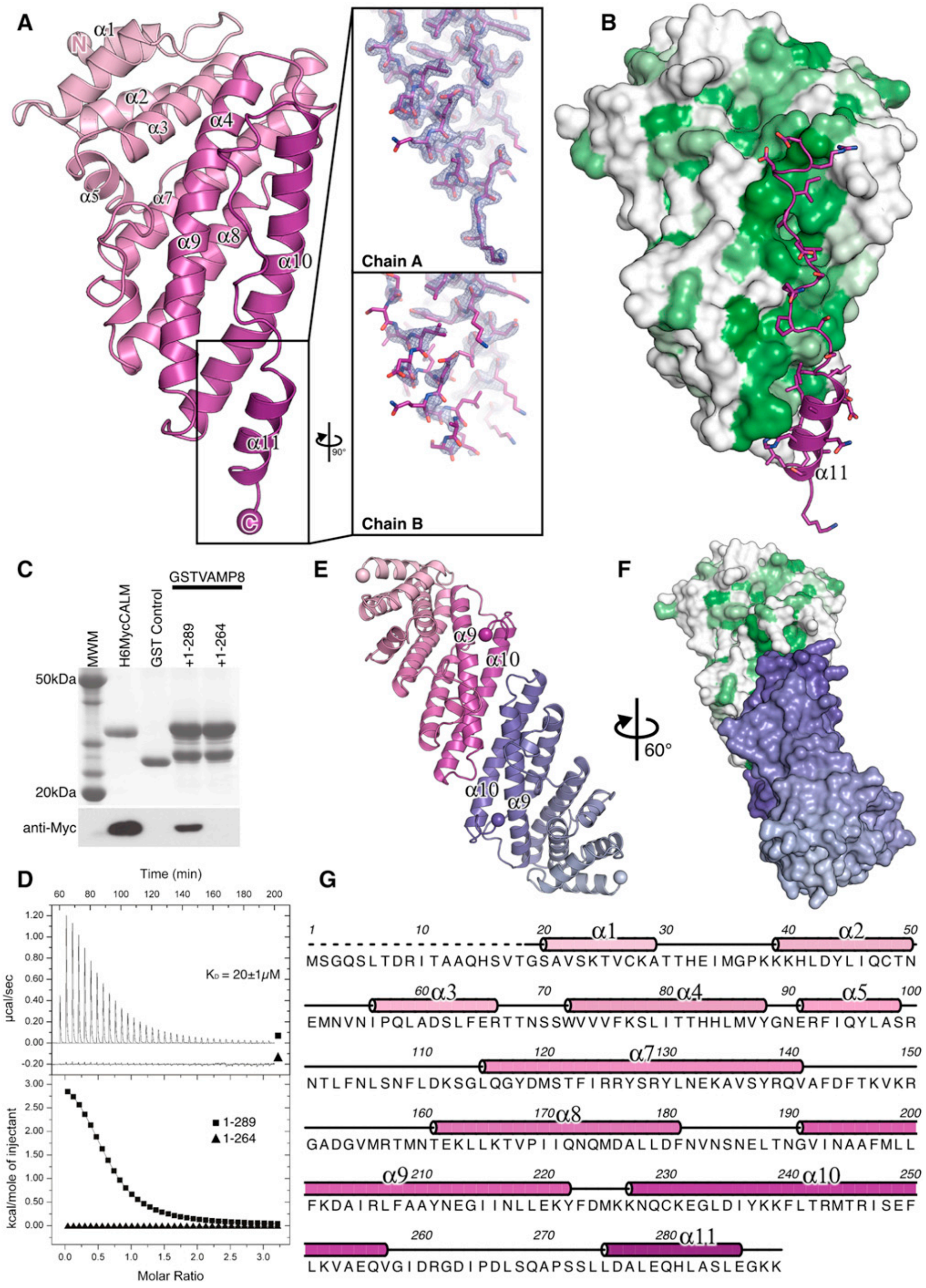

Figure 4. Structure of the $CALM_{ANTH}$ Domain

(A) Ribbon representation of the structure of the $CALM_{ANTH}$ domain colored from pink (residue 19) to purple (residue 288). Helices are numbered as in (Ford et al., 2001) with no α6. The insets show $2F_O–F_C$ electron density contoured at 1.2 σ for the well- and poorly-ordered α11 helix in the two $CALM_{ANTH}$ molecules in the asymmetric unit (designated chains A and B).

(B) Surface representation of helices α1-α10 of the $CALM_{ANTH}$ domain colored from high (dark green) to low (white) hydrophobicity oriented as in (A). The hydrophobic groove in which helix α11 and the ten preceeding residues sit can be clearly seen.

(C) GST pull-down expriments using GST, GSTVAMP8, and the $His_6MycCALM_{ANTH}$ proteins indicated. Top panel: Coomassie blue stained gel. Lower panel: western blot probed with anti-myc. Residues 1–289 but not 1–264 of $CALM_{ANTH}$ bound to GSTVAMP8.

(D) ITC quantitating the binding of residues 1–264 of $CALM_{ANTH}$ to VAMP8. $CALM_{ANTH(1-264)}$ (black triangles) showed no measurable binding to VAMP8 whereas WT $CALM_{ANTH(1-289)}$ bound with a K_D of 20 ± 1μM (black squares). Data for $CALM_{ANTH(1-264)}$ is offset by −0.2 μcal/sec for clarity.

(E) Structure of the $CALM_{ANTH(19-264)}$ dimer. One monomer is colored pink/purple and the other blue. The view is rotated by 60° around the vertical axis relative to (A).

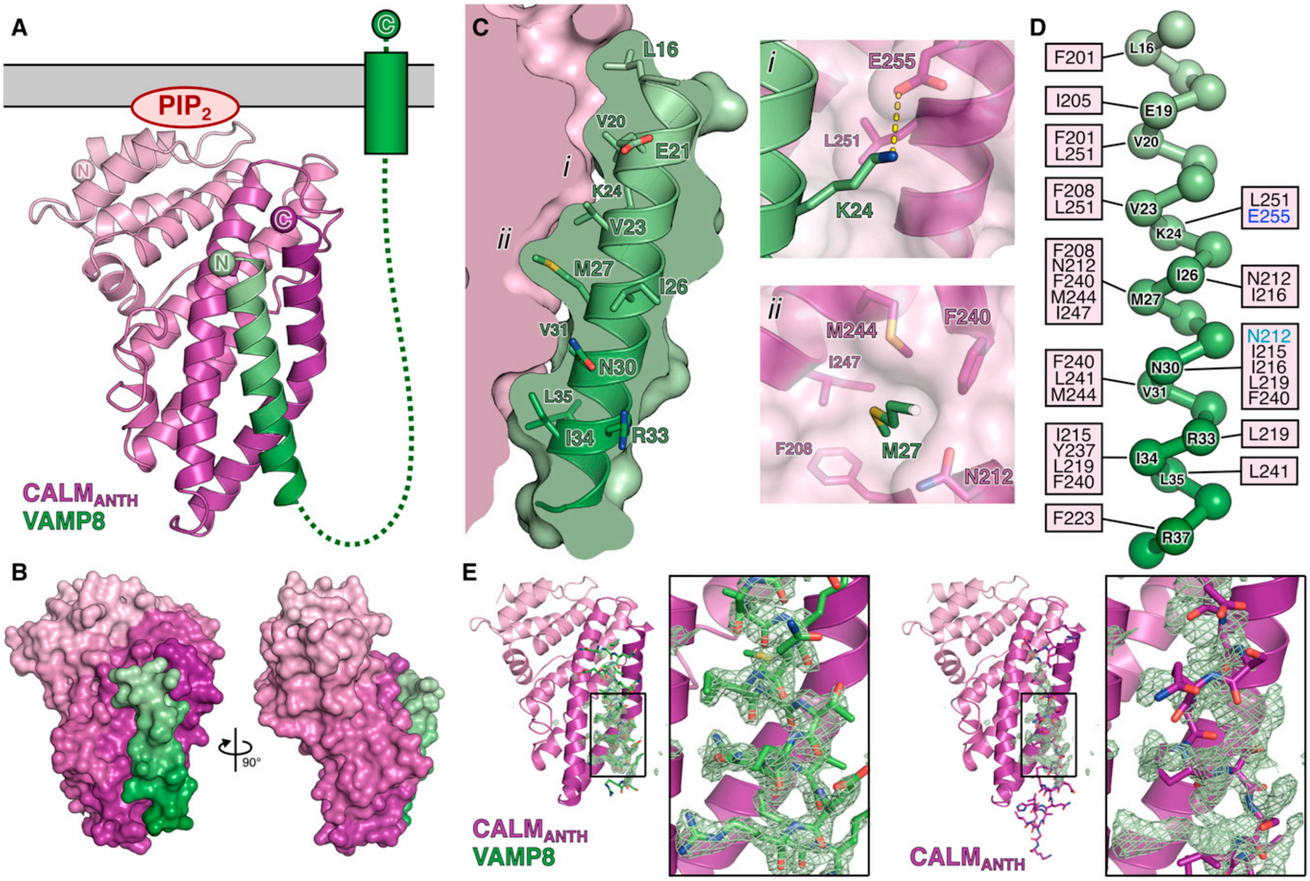

Figure 5. Structure of the $CALM_{ANTH(1\text{-}264)}$:$VAMP8_{(11\text{-}41)}$ Chimera

(A) Overall structure of the complex with $CALM_{ANTH}$ colored as in Figure 4 with VAMP8 colored from pale (residue 15) to dark (residue 37) green. The relative position of the membrane (gray bar) is inferred from the position of the PtdIns4,5P_2 (marked as PIP2) binding site on $CALM_{ANTH}$ as in 1HF8. The dotted line is a schematic representation of how the remainder of the cytoplasmic portion of VAMP8 domain connects its CALM binding helix to its transmembrane helix.

(B) Orthogonal views of the $CALM_{ANTH}$:VAMP8 complex shown in molecular surface representation colored as in (A).

(C) Spatial complementarity of the $CALM_{ANTH}$:VAMP8 interface with key side chains involved in the binding of $CALM_{ANTH}$ by VAMP8 shown. Molecular details of the interactions of the key residues (i) K24 and (ii) M27 are shown.

(D) Schematic representation of VAMP8 residues 15–38. $CALM_{ANTH}$ residues that make hydrophobic interactions with VAMP8 are labeled in black, those that make salt bridge interactions with VAMP8 are labeled in blue and those that make hydrogen bonds with VAMP8 are labeled in turquoise.

(E) The final refined VAMP8 helix (green sticks) is shown in unbiased (F_O–F_C) electron density contoured at 3.5 σ calculated before the addition of the helix to the model (left panel). The VAMP8 helix lies on the same face as, but differs significantly from, the orientation of helix α11 (purple sticks) in the unliganded $CALM_{ANTH}$ structure (right panel).

See also Figure S5.

the relevant portion of the VAMP's cytoplasmic domain could stretch up to 130Å. This is easily sufficient to allow a single CALM molecule, to simultaneously bind a VAMP8 molecule and a PtdIns4,5P_2 molecule from the same membrane.

In support of this model in which most or all of the portion of small R-SNARE VAMPs between their CALM binding helices and their transmembrane helices is unstructured, insertion of helix-disrupting proline residues in the predicted unstructured C-terminal half of the SNARE motif at positions Leu44 and Leu51 of VAMP8 does not inhibit its ability to bind CALM or to be endocytosed (Figures 7D–7F and S6). It does, however, prevent SNARE complex formation with SNAP23 and Syntaxin3 (Figure 7B), which relies on the VAMP being able to form a helix over the entire length of its SNARE motif. In contrast, introducing proline residues at positions Leu16 and Val23 in the CALM binding helix of VAMP8 blocks the interaction between the two

(F) Surface representation of the $CALM_{ANTH(1\text{-}264)}$ dimer with one monomer colored by hydrophobicity as in (B) and the other monomer colored blue, oriented as in (A). Formation of the dimer obscures the surface buried by helix α11 in $CALM_{ANTH(1\text{-}289)}$ which is proposed to be the VAMP8 binding site.

(G) Sequence of $CALM_{ANTH(1\text{-}289)}$. Secondary structure is shown above the sequence representing helices α1 to α11; α6 has been omitted as per (Ford et al., 2001).

See also Figure S4.

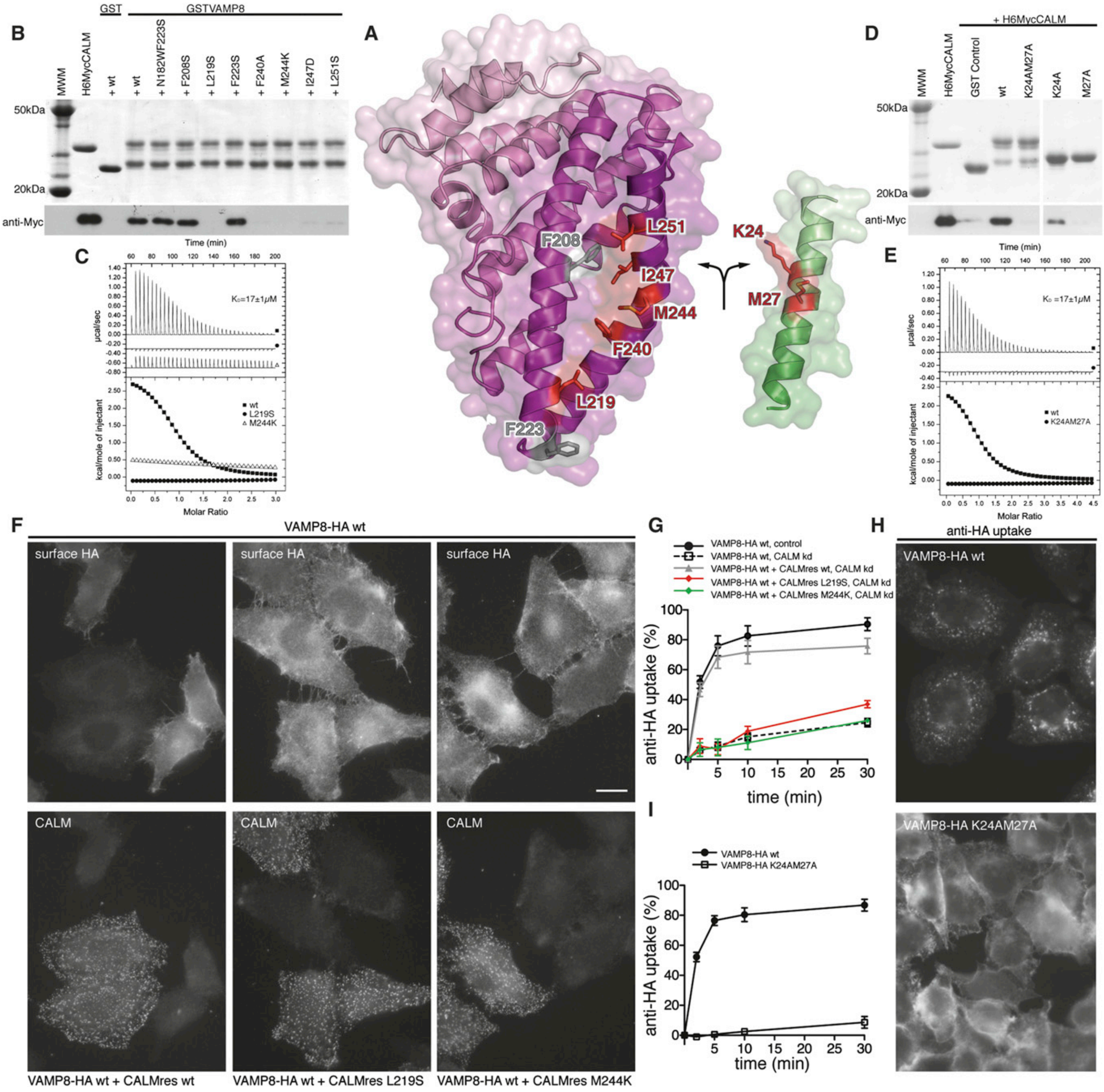

Figure 6. Mutation of Key Residues in the $CALM_{ANTH}$:VAMP8 Interface Abolishes Their Interaction In Vitro and the Endocytosis of VAMP8 In Vivo

(A) Structure of the $CALM_{ANTH}$:VAMP8 complex "opened out like a book" as indicated by the arrows. Residues whose mutation affect binding between CALM and VAMP8 are colored and labeled in red, while mutations that have no effect are colored and labeled in gray.

(B) Pull-down experiments using GSTVAMP8 and WT or mutant $His_6MycCALM_{ANTH}$ as indicated. Top panel: Coomassie blue stained gel. Lower panel: western blot probed with anti-myc. The mutations L219S, F240A, M244K, I247D, and L251S in $His_6MycCALM_{ANTH}$ abolished binding to VAMP8.

(C) ITC quantitating the binding of certain point mutant versions of $CALM_{ANTH}$ to VAMP8. The wild-type $CALM_{ANTH}$ binds with a K_D17 ± 1μM (black squares) whereas $CALM_{ANTH}$ L219S (black circles) and M244K (open triangles) showed no measurable interaction with VAMP8. Data for $CALM_{ANTH}$ L219S and M244K are translated by 0.3 μcal/s and −0.7 μcal/s respectively.

(D) GST pull-down experiments using $His_6MycCALM_{ANTH}$ and WT or mutant GSTVAMP8 fusion proteins as indicated. Top panel: Coomassie blue stained gel. Lower panel: western blot probed with anti-myc. Wt GSTVAMP8 bound $CALM_{ANTH}$ whereas the K24AM27A VAMP8 did not. The GSTVAMP8 K24A mutant interacted weakly with $CALM_{ANTH}$, however, the single M27A mutation of VAMP8 was sufficient to completely abolish the interaction with $CALM_{ANTH}$.

(E) ITC quantitating the binding of K24AM27A mutant version of VAMP8 to $CALM_{ANTH}$. Wt GSTVAMP8 bound wt $CALM_{ANTH}$ with a K_D17 ± 1μM (black squares) whereas GSTVAMP8 K24AM27A (black circles) showed no measurable interaction. Data for GSTVAMP8 K24AM27A is translated by −0.3 μcal/s for clarity.

proteins and prevents VAMP8 endocytosis as well as SNARE complex formation. These observations also are in line with the assertion made earlier that SNARE complex formation and the ability to be endocytosed are not linked, which disagrees with the interpretation of data in (Gordon et al., 2009).

DISCUSSION

The endocytic vesicles in most cells must incorporate the R-SNAREs that will allow them to fuse with their target endosome (likely VAMP8 and VAMP3 [Antonin et al., 2000; McMahon et al., 1993]). Alternatively, if they are to undergo immediate recycling without passing through an endosomal compartment, as has been proposed for neuronal synaptic vesicles, the vesicles need to be able to fuse with the plasma membrane, for which it is believed they use VAMP2 (Grote et al., 1995). Vesicles destined for endosomes must also return any SNAREs that have previously been used for fusion with the plasma membrane for reuse in subsequent vesicle/organelle fusion events. Such SNAREs include VAMP3 and VAMP8 for recycling back to the plasma membrane; VAMP7 and VAMP8 for endosome/lysosome biogenesis (Advani et al., 1998; Antonin et al., 2000; Pryor et al., 2004); VAMP8, VAMP7 and VAMP2 for the regulated secretion of lysosomes with the plasma membrane (reviewed in Chaineau et al., 2009); and VAMP4 for TGN-to-endosome transport (Steegmaier et al., 1999; Tran et al., 2007). VAMP7, which is active on late endocytic organelles (Advani et al., 1998; Pryor et al., 2004), has been shown to be endocytosed in an inactive cis-SNARE complex with a SNAP and a syntaxin family member (Pryor et al., 2008) through binding to the clathrin adaptor and ArfGAP Hrb. Transport in an inactive form prevents erroneous VAMP7-mediated fusion of endocytic vesicles with degradative late endosomes and lysosomes.

In contrast, we demonstrate that VAMP8, VAMP3, and VAMP2, which are required for the fusion of endocytic vesicles with their target early endosomes, are endocytosed in an uncomplexed and therefore essentially active form through their interaction with $CALM_{ANTH}$. However, since binding to CALM and participation in SNARE complexes are mutually exclusive processes, as both binding events utilize the same face of the VAMP, it would be more correct to state that VAMPs 8, 3, and 2 are endocytosed in only a potentially active form, as their conserved SNARE motifs are shielded by being bound to $CALM_{ANTH}$. Corecognition of PtdIns4,5P_2 markedly increases the strength of binding between CALM and a membrane containing both a small R-SNARE VAMP and PtdIns4,5P_2. In order for a small R-SNARE VAMP to bind to $CALM_{ANTH}$, helix 11 of the ANTH domain must be displaced. This dissociation will be facilitated by the fact that helix11 is only poorly associated with the rest of the ANTH domain (helices 1–10) (Figure 4) and may be further modulated by the C-terminal tail of CALM that follows helix11 binding to the clathrin terminal domains that are displayed on the underside of the polymeric clathrin lattice (Morgan et al., 2000). It should be noted that despite it being necessary to truncate $CALM_{ANTH}$ at residue 264 in order to obtain the structure of the $CALM_{ANTH}$:VAMP8 complex, the displacement of helix11 must have occurred in our biochemical assays as all were performed on residues 1–289 of $CALM_{ANTH}$.

Following scission of an endocytic clathrin-coated vesicle (CCV), the vesicle will uncoat by processes that include PtdIns4,5P_2 hydrolysis and clathrin cage disassembly. Once PtdIns4,5P_2 is hydrolyzed, the avidity effect generated by a single CALM molecule simultaneously binding PtdIns4,5P_2 and a small R-SNARE VAMP will vanish leaving only the weak, transient R-SNARE:CALM interaction. The two proteins will therefore quickly dissociate. In order to minimise the energetically unfavorable situation of the hydrophobic face of an amphipathic helix being exposed to the aqueous environment, the N-terminal half of the SNARE motif of the short R-SNAREs, while still remaining as a helix, switches to lying on the surface of the vesicle membrane (Ellena et al., 2009). In the case of other SNAREs such as the syntaxins and longin domain SNAREs, the SNARE motifs can be shielded from the aqueous environment by binding back on their N-terminal regulatory domains, which are not present in the small R-SNAREs. On reaching its final destination, the VAMP will now form tight, energetically favorable complexes with its cognate Q-SNAREs on an endosome and so drive fusion between the endocytic vesicle and endosomal membranes. Thus in the cell, the SNARE motif of a small R-SNARE is never free but interacts with either CALM, a membrane surface or is part of a cognate SNARE complex (Figure 7G).

Nonsense point mutations in CALM are responsible for hematopoietic and iron metabolism abnormalities, growth retardation, and shortened life span in fit1 mice (Klebig et al., 2003), the strongest phenotypes resulting from premature translational termination in the $CALM_{ANTH}$ domain. The CALM/PICALM gene has also been directly implicated in alterations in cognitive function with increasing age (Mengel-From et al., 2011) in risk of developing Alzheimer's disease (Harold et al., 2009) and in modifying the toxicity of Aβ in a yeast, *C. elegans* and primary rat cortical neuron models (Treusch et al., 2011). These pathophysiological effects could, until now, only be explained by the role of CALM

(F) Cells expressing either VAMP8-HA alone, or VAMP8-HA plus Myc-tagged siRNA-resistant CALM (CALMres: wt, L219S, or M244K) were mixed together and endogenous CALM was depleted by siRNA treatment. The cells were then fixed without permeabilization and labeled with anti-HA antibody, and then permeabilized and labeled with anti-CALM. The scale bar represents 20 μm.

(G) Endocytosis of anti-HA in cells coexpressing VAMP8-HA and siRNA-resistant wild-type, L219S or M244K mutant myc-tagged CALM. Antibody was bound to the cells at 4°C, then the cells were warmed to 37°C for 2–30 min and antibody remaining at the cell surface was quantified by flow cytometry. Each point is derived from at least 3 separate experiments; the error bars show the SEM. Expression of the CALM construct rescues the knockdown phenotype, but expression of the two CALM mutants does not.

(H) Localization of anti-HA in cells expressing wild-type or K24AM27A mutant VAMP8-HA. The cells were allowed to endocytose the antibody for 40 min, then processed for immunofluorescence. Unlike the cells expressing wild-type VAMP8, the cells expressing the mutant have retained the antibody on the plasma membrane. The scale bar represents 20 μm.

(I) Endocytosis of anti-HA in cells expressing wild-type or K24AM27A mutant VAMP8-HA, using the flow cytometry assay described in Figure S1B. There is negligible endocytosis of the mutant construct.

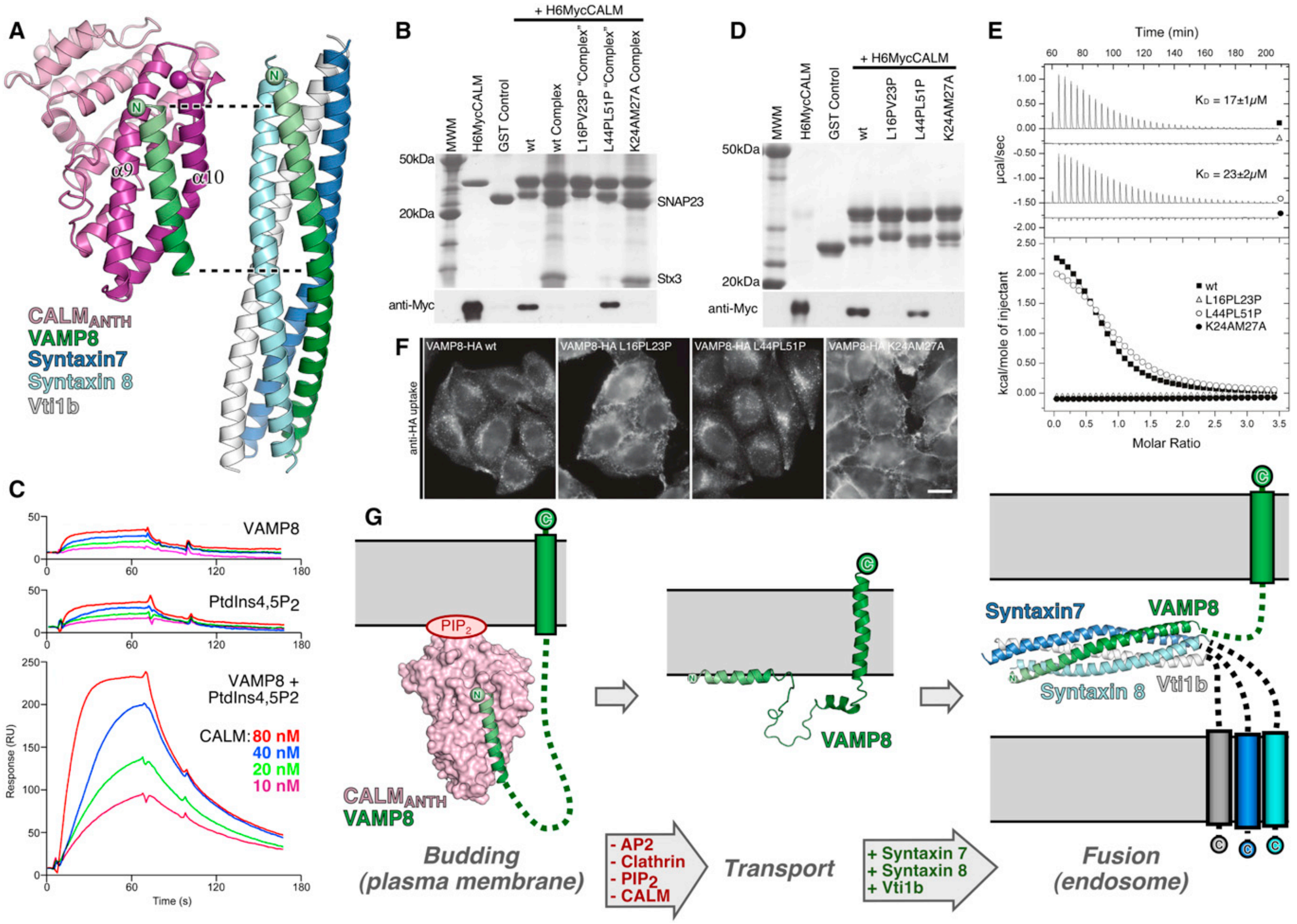

Figure 7. Binding of $CALM_{ANTH}$ and SNARE Complex Formation by VAMP8 Are Mutually Exclusive

(A) Comparison of the mode of VAMP8 binding to $CALM_{ANTH}$ and to Syntaxin7:Syntaxin8:Vti1b (PDB 1GL2) (Antonin et al., 2002) made by superimposing the VAMP8 residues 15–39 from the two complexes. VAMP8 adopts the same superhelical twist in both structures and helices α9 and α10 of $CALM_{ANTH}$ correspond to the helices of Syntaxin 8 and Syntaxin 7, respectively.

(B) Formation of GSTVAMP8:SNAP23:Synatxin3(195–253) SNARE complexes (see Supplemental Information) and their binding to $CALM_{ANTH}$. Pull-down experiments using $His_6MycCALM_{ANTH}$ and the GST fusion proteins indicated. Top panel: Coomassie blue stained gel. Lower panel: western blot probed with anti-myc. SNARE complexes formed with GSTVAMP8 wt and K24AM27A but not with GSTVAMP8 L16PV23P or L44PL51P. Neither the wt VAMP8 SNARE complex nor the K24AM27A SNARE complex bound $CALM_{ANTH}$ indicating that SNARE complex formation and $CALM_{ANTH}$ binding shown are mutually exclusive events.

(C) Avidity of CALM binding to PtdIns4,$5P_2$ and VAMP8. Shown are the sensorgrams for the concentration dependent binding of CALM to membranes with captured VAMP8 (top: calculated K_D0.9 ± 0.15 μM), membranes with PtdIns4,$5P_2$ (middle: calculated K_D1.9 ± 0.45 μM) and membranes with both, PtdIns4,$5P_2$ and captured VAMP8 (bottom: calculated K_D0.17 ± 0.03 μM). Given are the mean values and SD of four independent measurements The affinity of CALM increases by 8.5 fold over the average K_D for the two ligands when VAMP8 and PtdIns4,$5P_2$ are bound simultaneously.

(D) Pull-down experiments using $His_6MycCALM_{ANTH}$ and wt and mutant GSTVAMP8 fusion proteins as indicated. Top panel: Coomassie blue stained gel. Lower panel: western blot probed with anti-myc. The GSTVAMP8 mutants L16PV23P and K24AM27A (see also Figure 6) do not bind $CALM_{ANTH}$. However, GSTVAMP8 L44PL51P bound $CALM_{ANTH}$ with a similar strength to wt GSTVAMP8.

(E) ITC quantitating the binding of point mutated versions of VAMP8 to $CALM_{ANTH}$. The binding of wt $CALM_{ANTH}$ and GSTVAMP8 L44PL51P (open circles) was comparable to that of wt $CALM_{ANTH}$ and wt GSTVAMP8 (black squares) (K_Ds of 23 ± 2μM and 17 ± 1μM respectively). However, GSTVAMP8 L16PV23P (open triangles) and GSTVAMP8 K24AM27A (black circles) both showed no measurable interaction with $CALM_{ANTH}$. Data for GSTVAMP8 L16PL23P, L44PL51P and K24AM27A are translated by −0.3 μcal/s, −1.5 μcal/s and −1.9 μcal/s respectively for clarity.

(F) Localization of anti-HA in cells expressing different VAMP8-HA constructs. The cells were allowed to endocytose the antibody for 40 min, then processed for immunofluorescence. The cells expressing wt VAMP8 and the L44PL51P mutant have endocytosed the antibody, but the cells expressing the L16PV23P mutant have mainly retained the antibody on the plasma membrane similar to the K24AM27A mutant. The scale bar represents 20 μm.

(G) Schematic representation of the model of VAMP8 trafficking from the plasma membrane. The clathrin adaptor CALM binds simultaneously to the R-SNARE VAMP8 and PtdIns4,$5P_2$ (labeled PIP_2) at the plasma membrane. CALM is released from the surface of an endocytosed vesicle when PtdIns4,$5P_2$ hydrolyzed and the clathrin cage disassembled and the hydrophobic CALM-binding helix of VAMP8 now "lies" on the vesicle's surface (Ellena et al., 2009). Finally VAMP8 forms a trans-SNARE complex with its cognate SNAREs on an early endosome to drive vesicule fusion. Thus throughout the interaction of the hydrophobic VAMP8 SNARE motif with the aqueous environment is minimized.

in linking clathrin to the PtdIns4,5P_2-containing membrane during endocytosis, a role replicated by all clathrin adaptors. The work presented here raises the possibility that such effects are related to CALM's ability to directly drive the endocytosis of the small R-SNAREs VAMP8, VAMP3, and VAMP2, since failure to correctly transport these small R-SNARE VAMPs to early endosomes as a result of a reduction in CALM levels would perturb subsequent trafficking of a wide variety of proteins through the endocytic pathway. In the case of Alzheimer's especially it appears that any alterations in vesicle trafficking give rise to increased levels of Aβ production, presumably by modifying the localization of Amyloid Precursor Protein (APP) or that of its processing proteases (α-, β-, and γ secretases) (Burgos et al., 2010; Lee et al., 2008; Sannerud and Annaert, 2009). Further, it has been documented that a major pathway for clearance of Aß from the brain parenchyma is endocytosis by various cell types including astrocytes, microglia, and endothelial cells. The latter may further mediate Aß clearance from the brain through the blood-brain-barrier by transcytosis, and it is of note that these cells were shown to possess the highest levels of CALM expression in the brain (Baig et al., 2010; Bu, 2009). This provides another possible explanation for why CALM has been linked to risk of Alzheimer's disease since CALM should play a key role in this endocytic process by selecting the R-SNAREs needed both for endocytic vesicle/endosome fusion and for subsequent fusion events along the endocytic pathway.

In summary, we have provided insight into the molecular mechanism by which the post-Golgi small R-SNAREs bind specifically to the ubiquitously and highly expressed endocytic clathrin adaptor CALM, recently confirmed in Koo et al. (2011). This ensures that the SNAREs required for fusion with the endosomal system and for subsequent trafficking steps are actively selected into endocytic CCVs. This ability to mediate the endocytosis of small R-SNARE VAMPs from the cell's limiting membrane provides a possible explanation for the association of CALM with a variety of both neurological and other disorders.

EXPERIMENTAL PROCEDURES

For a list of the constructs and full materials and methods used in this study, please see the Extended Experimental Procedures.

Cell Biology

HeLaM cells were used for all experiments. For most experiments, the cells were stably transfected with HA-tagged wild-type or mutant VAMPs (2, 3, and 8), and cell lines were selected that expressed the construct at low levels so as not to saturate sorting machinery. For some experiments, the cells were additionally transfected with myc-tagged, siRNA-resistant wild-type or mutant CALM, and cell lines were selected that expressed the tagged CALM at similar levels to endogenous CALM. The steady state localization of the constructs and other proteins, both under control conditions and after siRNA-mediated knockdowns, was observed by immunofluorescence microscopy. Endocytosis kinetics were measured using either a flow cytometry-based antibody uptake assay for the tagged VAMPs, or a radioiodinated ligand uptake assay for other surface receptors. Details of transfection and knockdown conditions, immunolabelling, and endocytosis assays are all described in Supplemental Information.

Protein Expression and Purification

All recombinant proteins were expressed in BL21(DE3) pLysS *E. coli* for 16 hr at 22°C after induction with 0.2 mM IPTG at 37°C and purified by standard procedures on glutathione sepharose and/or Ni^{2+}-NTA agarose as appropriate. GST-tagged proteins were eluted with free glutathione or by thrombin cleavage of the GST-tag while fusion proteins were bound to the beads. His_6-tagged proteins were eluted with buffer containing 300mM imidazole. All proteins were subsequently purified by S200 gel filtration.

Recombinant Protein Pull Downs

SNARE:adaptor interactions were tested using varying concentrations of GST-tagged SNAREs and relevant His_6myc-tagged prey proteins. SNARE complexes were made with a 3-fold excess of SNAP23 and syntaxin3 to GSTVAMP8 and purified by GST sepharose and gel filtration. SNARE complex:adaptor interactions were tested using 2 nmoles of 1:1:1 GST-tagged complex incubated with His_6myc-$CALM_{ANTH}$. All binding experiments were carried out in 1 ml of buffer supplemented with 30 μl glutathione beads and incubated with constant agitation at 4°C. The supernatant removed and the beads washed with 1 ml of buffer, three times. Bound proteins were analyzed by SDS-PAGE and western blots probed with anti-myc antibody (9E10 Santa Cruz Biotechnologies).

Isothermal Titration Calorimetry

Due to yield and solubility issues, GST-tagged SNAREs were used. A VPITC machine (GE Healthcare) was used to titrate 37 injections of 4–8 μl 2 mM His_6Myc$CALM_{ANTH}$ or GST cleaved Epsin1_{ENTH} proteins into 0.15 mM SNARE proteins. Titration curves were fitted using ORIGIN software. Figures show a representative example of each experiment with the K_D and associated confidence of the fit (SEM) shown; n = 0.9-1.1. K_Ds quoted in the text are the average of all runs.

Surface Plasmon Resonance

Liposome-based SPR was carried out using a Biacore 3000 (GE Healthcare) with GST cleaved $CALM_{ANTH}$ and Epsin1_{ENTH} adaptor proteins as analytes and PC/PE liposomes supplemented with 5% PtdIns4,5P_2 and/or His_{12}-tagged VAMP8 attached via 5% DGS-NTA(Ni). The binding was monitored during one minute injections at 50 μl/min at concentrations ranging from 10 nM to 50 μM. The kinetic parameters were calculated after background (PC/PE binding) subtraction.

Crystallization, Data Collection, Structure Solution, and Refinement

Crystals of $CALM_{ANTH\,(1\text{-}289)}$, $CALM_{ANTH(1\text{-}264)}$, and $CALM_{ANTH(1\text{-}264)}$: $VAMP8_{(11\text{-}41)}$ were grown in sitting drops at 16°C using GST fusion proteins form which the GST had been cleaved. $CALM_{ANTH(1\text{-}289)}$ was equilibrated against reservoirs containing 100 mM Bis Tris propane, 200 mM sodium malonate, 20% (w/v) PEG 3350; $CALM_{ANTH(1\text{-}264)}$ was equilibrated against reservoirs containing 15% v/v ethanol, 100 mM imidazole (pH 8.0), 200 mM $MgCl_2$; $CALM_{ANTH}$:VAMP8 was equilibrated against reservoirs containing 100 mM phosphate-citrate (pH 4.2), 200 mM NaCl, 50% (v/v) PEG200. All structures were solved by molecular replacement using previously published ANTH domain of CALM as a starting model (1HF8). Detailed descriptions of structure determination, refinement, and structural analysis can be found in Supplemental Information. Structure factors and final refined coordinates have been deposited in the PDB with accession codes 3ZYK ($CALM_{ANTH}$), 3ZYL ($CALM_{ANTH(1\text{-}264)}$), and 3ZYM ($CALM_{ANTH(1\text{-}264)}$:$VAMP8_{(11\text{-}41)}$).

SUPPLEMENTAL INFORMATION

Supplemental Information includes Extended Experimental Procedures, one table, and six figures and can be found with this article online at doi:10.1016/j.cell.2011.10.038.

ACKNOWLEDGMENTS

We thank the beamline scientists at Diamond Light Source plc and Janet Deane for assistance with data collection; and Susan Lea and Steven Johnson (University of Oxford) for assistance with MALS experiments. We also thank Bazbek Davletov (MRCLMB) for gifts of VAMP2, SNAP23, and syntaxin3 DNA. D.J.O. and M.S.R. are funded by Wellcome Trust fellowships; A.A.P. by an MRC CDF (G120/952); and S.C.G. is an 1851 Research Fellow. S.H. is

funded by a grant from the German Science Foundation (SFB 635). The protein chemistry, structure determinations and biophysical assays were carried out by S.E.M., S.C.G., S.H., and D.J.O.; the cell biology by D.A.S., M.S.R., and A.A.P.

Received: January 26, 2011
Revised: July 7, 2011
Accepted: October 27, 2011
Published: November 23, 2011

REFERENCES

Advani, R.J., Bae, H.R., Bock, J.B., Chao, D.S., Doung, Y.C., Prekeris, R., Yoo, J.S., and Scheller, R.H. (1998). Seven novel mammalian SNARE proteins localize to distinct membrane compartments. J. Biol. Chem. *273*, 10317–10324.

Antonin, W., Fasshauer, D., Becker, S., Jahn, R., and Schneider, T.R. (2002). Crystal structure of the endosomal SNARE complex reveals common structural principles of all SNAREs. Nat. Struct. Biol. *9*, 107–111.

Antonin, W., Holroyd, C., Tikkanen, R., Honing, S., and Jahn, R. (2000). The R-SNARE endobrevin/VAMP-8 mediates homotypic fusion of early endosomes and late endosomes. Mol. Biol. Cell *11*, 3289–3298.

Baig, S., Joseph, S.A., Tayler, H., Abraham, R., Owen, M.J., Williams, J., Kehoe, P.G., and Love, S. (2010). Distribution and expression of picalm in Alzheimer disease. J. Neuropathol. Exp. Neurol. *69*, 1071–1077.

Bao, H., Daniels, R.W., MacLeod, G.T., Charlton, M.P., Atwood, H.L., and Zhang, B. (2005). AP180 maintains the distribution of synaptic and vesicle proteins in the nerve terminal and indirectly regulates the efficacy of Ca2+-triggered exocytosis. J. Neurophysiol. *94*, 1888–1903.

Bock, J.B., Matern, H.T., Peden, A.A., and Scheller, R.H. (2001). A genomic perspective on membrane compartment organization. Nature *409*, 839–841.

Bonifacino, J.S., and Traub, L.M. (2003). Signals for sorting of transmembrane proteins to endosomes and lysosomes. Annu. Rev. Biochem. *72*, 395–447.

Bu, G. (2009). Apolipoprotein E and its receptors in Alzheimer's disease: pathways, pathogenesis and therapy. Nat. Rev. Neurosci. *10*, 333–344.

Burgos, P.V., Mardones, G.A., Rojas, A.L., daSilva, L.L., Prabhu, Y., Hurley, J.H., and Bonifacino, J.S. (2010). Sorting of the Alzheimer's disease amyloid precursor protein mediated by the AP-4 complex. Dev. Cell *18*, 425–436.

Burston, H.E., Maldonado-Baez, L., Davey, M., Montpetit, B., Schluter, C., Wendland, B., and Conibear, E. (2009). Regulators of yeast endocytosis identified by systematic quantitative analysis. J. Cell Biol. *185*, 1097–1110.

Bushlin, I., Petralia, R.S., Wu, F., Harel, A., Mughal, M.R., Mattson, M.P., and Yao, P.J. (2008). Clathrin assembly protein AP180 and CALM differentially control axogenesis and dendrite outgrowth in embryonic hippocampal neurons. J. Neurosci. *28*, 10257–10271.

Chaineau, M., Danglot, L., and Galli, T. (2009). Multiple roles of the vesicular-SNARE TI-VAMP in post-Golgi and endosomal trafficking. FEBS Lett. *583*, 3817–3826.

Dreyling, M.H., Martinez-Climent, J.A., Zheng, M., Mao, J., Rowley, J.D., and Bohlander, S.K. (1996). The t(10;11)(p13;q14) in the U937 cell line results in the fusion of the AF10 gene and CALM, encoding a new member of the AP-3 clathrin assembly protein family. Proc. Natl. Acad. Sci. USA *93*, 4804–4809.

Ellena, J.F., Liang, B., Wiktor, M., Stein, A., Cafiso, D.S., Jahn, R., and Tamm, L.K. (2009). Dynamic structure of lipid-bound synaptobrevin suggests a nucleation-propagation mechanism for trans-SNARE complex formation. Proc. Natl. Acad. Sci. USA *106*, 20306–20311.

Fasshauer, D., Otto, H., Eliason, W.K., Jahn, R., and Brunger, A.T. (1997). Structural changes are associated with soluble N-ethylmaleimide-sensitive fusion protein attachment protein receptor complex formation. J. Biol. Chem. *272*, 28036–28041.

Fasshauer, D., Sutton, R.B., Brunger, A.T., and Jahn, R. (1998). Conserved structural features of the synaptic fusion complex: SNARE proteins reclassified as Q- and R-SNAREs. Proc. Natl. Acad. Sci. USA *95*, 15781–15786.

Fiebig, K.M., Rice, L.M., Pollock, E., and Brunger, A.T. (1999). Folding intermediates of SNARE complex assembly. Nat. Struct. Biol. *6*, 117–123.

Ford, M.G., Pearse, B.M., Higgins, M.K., Vallis, Y., Owen, D.J., Gibson, A., Hopkins, C.R., Evans, P.R., and McMahon, H.T. (2001). Simultaneous binding of PtdIns(4,5)P2 and clathrin by AP180 in the nucleation of clathrin lattices on membranes. Science *291*, 1051–1055.

Gordon, D.E., Mirza, M., Sahlender, D.A., Jakovleska, J., and Peden, A.A. (2009). Coiled-coil interactions are required for post-Golgi R-SNARE trafficking. EMBO Rep. *10*, 851–856.

Grote, E., Hao, J.C., Bennett, M.K., and Kelly, R.B. (1995). A targeting signal in VAMP regulating transport to synaptic vesicles. Cell *81*, 581–589.

Harel, A., Wu, F., Mattson, M.P., Morris, C.M., and Yao, P.J. (2008). Evidence for CALM in directing VAMP2 trafficking. Traffic *9*, 417–429.

Harold, D., Abraham, R., Hollingworth, P., Sims, R., Gerrish, A., Hamshere, M.L., Pahwa, J.S., Moskvina, V., Dowzell, K., Williams, A., et al. (2009). Genome-wide association study identifies variants at CLU and PICALM associated with Alzheimer's disease. Nat. Genet. *41*, 1088–1093.

Hazzard, J., Sudhof, T.C., and Rizo, J. (1999). NMR analysis of the structure of synaptobrevin and of its interaction with syntaxin. J. Biomol. NMR *14*, 203–207.

Hong, W. (2005). SNAREs and traffic. Biochim. Biophys. Acta *1744*, 493–517.

Huang, F., Khvorova, A., Marshall, W., and Sorkin, A. (2004). Analysis of clathrin-mediated endocytosis of epidermal growth factor receptor by RNA interference. J. Biol. Chem. *279*, 16657–16661.

Jahn, R., and Scheller, R.H. (2006). SNAREs–engines for membrane fusion. Nat. Rev. Mol. Cell Biol. *7*, 631–643.

Klebig, M.L., Wall, M.D., Potter, M.D., Rowe, E.L., Carpenter, D.A., and Rinchik, E.M. (2003). Mutations in the clathrin-assembly gene Picalm are responsible for the hematopoietic and iron metabolism abnormalities in fit1 mice. Proc. Natl. Acad. Sci. USA *100*, 8360–8365.

Kloepper, T.H., Kienle, C.N., and Fasshauer, D. (2007). An elaborate classification of SNARE proteins sheds light on the conservation of the eukaryotic endomembrane system. Mol. Biol. Cell *18*, 3463–3471.

Koo, S.J., Markovic, S., Puchkov, D., Mahrenholz, C.C., Beceren-Braun, F., Maritzen, T., Dernedde, J., Volkmer, R., Oschkinat, H., and Haucke, V. (2011). SNARE motif-mediated sorting of synaptobrevin by the endocytic adaptors clathrin assembly lymphoid myeloid leukemia (CALM) and AP180 at synapses. Proc. Natl. Acad. Sci. USA *108*, 13540–13545.

Krissinel, E., and Henrick, K. (2007). Inference of macromolecular assemblies from crystalline state. J. Mol. Biol. *372*, 774–797.

Lee, J., Retamal, C., Cuitino, L., Caruano-Yzermans, A., Shin, J.E., van Kerkhof, P., Marzolo, M.P., and Bu, G. (2008). Adaptor protein sorting nexin 17 regulates amyloid precursor protein trafficking and processing in the early endosomes. J. Biol. Chem. *283*, 11501–11508.

Lewis, M.J., Nichols, B.J., Prescianotto-Baschong, C., Riezman, H., and Pelham, H.R. (2000). Specific retrieval of the exocytic SNARE Snc1p from early yeast endosomes. Mol. Biol. Cell *11*, 23–38.

Lindner, R., and Ungewickell, E. (1992). Clathrin-associated proteins of bovine brain coated vesicles. An analysis of their number and assembly-promoting activity. J. Biol. Chem. *267*, 16567–16573.

Mancias, J.D., and Goldberg, J. (2007). The transport signal on Sec22 for packaging into COPII-coated vesicles is a conformational epitope. Mol. Cell *26*, 403–414.

Martinez-Arca, S., Rudge, R., Vacca, M., Raposo, G., Camonis, J., Proux-Gillardeaux, V., Daviet, L., Formstecher, E., Hamburger, A., Filippini, F., et al. (2003). A dual mechanism controlling the localization and function of exocytic v-SNAREs. Proc. Natl. Acad. Sci. USA *100*, 9011–9016.

McMahon, H.T., Ushkaryov, Y.A., Edelmann, L., Link, E., Binz, T., Niemann, H., Jahn, R., and Sudhof, T.C. (1993). Cellubrevin is a ubiquitous tetanus-toxin substrate homologous to a putative synaptic vesicle fusion protein. Nature *364*, 346–349.

Mengel-From, J., Christensen, K., McGue, M., and Christiansen, L. (2011). Genetic variations in the CLU and PICALM genes are associated with cognitive function in the oldest old. Neurobiol. Aging *32*, e7–e11.

Miller, S.E., Collins, B.M., McCoy, A.J., Robinson, M.S., and Owen, D.J. (2007). A SNARE-adaptor interaction is a new mode of cargo recognition in clathrin-coated vesicles. Nature *450*, 570–574.

Morgan, J.R., Prasad, K., Hao, W., Augustine, G.J., and Lafer, E.M. (2000). A conserved clathrin assembly motif essential for synaptic vesicle endocytosis. J. Neurosci. *20*, 8667–8676.

Mossessova, E., Bickford, L.C., and Goldberg, J. (2003). SNARE selectivity of the COPII coat. Cell *114*, 483–495.

Nonet, M.L., Holgado, A.M., Brewer, F., Serpe, C.J., Norbeck, B.A., Holleran, J., Wei, L., Hartwieg, E., Jorgensen, E.M., and Alfonso, A. (1999). UNC-11, a Caenorhabditis elegans AP180 homologue, regulates the size and protein composition of synaptic vesicles. Mol. Biol. Cell *10*, 2343–2360.

Owen, D.J., Collins, B.M., and Evans, P.R. (2004). Adaptors for clathrin coats: structure and function. Annu. Rev. Cell Dev. Biol. *20*, 153–191.

Peden, A.A., Park, G.Y., and Scheller, R.H. (2001). The Di-leucine motif of vesicle-associated membrane protein 4 is required for its localization and AP-1 binding. J. Biol. Chem. *276*, 49183–49187.

Pryor, P.R., Jackson, L., Gray, S.R., Edeling, M.A., Thompson, A., Sanderson, C.M., Evans, P.R., Owen, D.J., and Luzio, J.P. (2008). Molecular basis for the sorting of the SNARE VAMP7 into endocytic clathrin-coated vesicles by the ArfGAP Hrb. Cell *134*, 817–827.

Pryor, P.R., Mullock, B.M., Bright, N.A., Lindsay, M.R., Gray, S.R., Richardson, S.C., Stewart, A., James, D.E., Piper, R.C., and Luzio, J.P. (2004). Combinatorial SNARE complexes with VAMP7 or VAMP8 define different late endocytic fusion events. EMBO Rep. *5*, 590–595.

Sannerud, R., and Annaert, W. (2009). Trafficking, a key player in regulated intramembrane proteolysis. Semin. Cell Dev. Biol. *20*, 183–190.

Sharpe, H.J., Stevens, T.J., and Munro, S. (2010). A comprehensive comparison of transmembrane domains reveals organelle-specific properties. Cell *142*, 158–169.

Steegmaier, M., Klumperman, J., Foletti, D.L., Yoo, J.S., and Scheller, R.H. (1999). Vesicle-associated membrane protein 4 is implicated in trans-Golgi network vesicle trafficking. Mol. Biol. Cell *10*, 1957–1972.

Sutton, R.B., Fasshauer, D., Jahn, R., and Brunger, A.T. (1998). Crystal structure of a SNARE complex involved in synaptic exocytosis at 2.4 A resolution. Nature *395*, 347–353.

Takamori, S., Holt, M., Stenius, K., Lemke, E.A., Gronborg, M., Riedel, D., Urlaub, H., Schenck, S., Brugger, B., Ringler, P., et al. (2006). Molecular anatomy of a trafficking organelle. Cell *127*, 831–846.

Tran, T.H., Zeng, Q., and Hong, W. (2007). VAMP4 cycles from the cell surface to the trans-Golgi network via sorting and recycling endosomes. J. Cell Sci. *120*, 1028–1041.

Treusch, S., Hamamichi, S., Goodman, J.L., Matlack, K.E., Chung, C.Y., Baru, V., Shulman, J.M., Parrado, A., Bevis, B.J., Valastyan, J.S., et al. (2011). Functional links between abeta toxicity, endocytic trafficking, and Alzheimer's disease risk factors in yeast. Science. Published online October 27, 2011. 10.1126/science.1213210.

Yao, P.J., Zhang, P., Mattson, M.P., and Furukawa, K. (2003). Heterogeneity of endocytic proteins: distribution of clathrin adaptor proteins in neurons and glia. Neuroscience *121*, 25–37.

EXTENDED EXPERIMENTAL PROCEDURES

Constructs Used in This Study

pGEX 4T2 VAMP8$_{1-76}$ (mouse) and mutants thereof, pGEX 4T2 VAMP8$_{(1-31)}$, pGEX 4T2 VAMP8$_{(1-41)}$, pGEX 4T2 VAMP8$_{(16-41)}$, pGEX 4T2 VAMP8$_{(10-41)}$, pGEX 4T2 VAMP3$_{(1-81)}$ (human), pGEX 4T2 VAMP2$_{(1-96)}$ (human), pGEX 4T1 SNAP23 (human), pGEX 4T1syntaxin3$_{(195-253)}$ (human), pGEX 4T2 CALM$_{ANTH(1-289)}$ (rat), pMWHis$_6$Myc CALM$_{ANTH(1-289)}$, pGEX 4T2 CALM$_{ANTH(1-264)}$, pMWHis$_6$Myc AP180$_{ANTH(1-304)}$ (mouse), pGEX 4T1 Epsin$_{ENTH(1-163)}$ (human), pGEX 4T2 CALM$_{ANTH(1-289)}$:VAMP8$_{(1-76)}$, pGEX CALM$_{ANTH(1-264)}$:VAMP8$_{(11-41)}$, pGEX 4T2 VAMP8$_{(1-76)}$His$_{12}$, pLXIN VAMP8 and mutants thereof, pLXIN VAMP3 and mutants thereof, pLXIN VAMP2 and mutants thereof.

pBMN-CALM (made siRNA resistant by the introduction of four silent point mutations in the siRNA oligo binding site) wild-type and mutants Leu219Ser or Met244Lys were derived from IMAGE clone 5537605 into which a myc tag had been inserted in a nonconserved region (position 1308 bp).

Cell Biology

For expression of HA-tagged VAMP constructs, HeLa M cells were infected with retrovirus using the Phoenix system, and cell lines were selected with 0.5 mg/ml G418, as previously described (Gordon et al., 2009). Endogenous CALM was depleted with an siRNA (CALM oligo 5; 5′-ACAGTTGGCAGACAGTTTA) (Dharmacon) at a concentration of 20 nM for 72 hr. For the rescue experiments, HA-tagged VAMP8-expressing cells were further transduced with constructs expressing myc-tagged siRNA-resistant CALM (both wild-type and mutant), and selected with 0.2 mg/ml hygromycin B together with 0.5 mg/ml G418.

Primary antibodies used for immunofluorescence and western blotting included a commercial goat polyclonal antibody against CALM (Santa Cruz), commercial mouse monoclonal antibodies against the HA epitope (Covance) and the myc eptiope (Millipore), in-house rabbit polyclonal antibodies against VAMP8 and VAMP3, and (for loading controls) mouse monoclonal antibodies against syntaxin4 and the AP-2 α subunit (both from BD Biosciences). For labeling HA-tagged VAMP8 on the plasma membrane, cells were fixed with 4% paraformaldehyde and labeled with mouse anti-HA, then permeabilized with 0.1% Triton X-100 and labeled with goat anti-CALM (Santa Cruz). For visualizing antibody uptake by immunofluorescence, cells were allowed to endocytose anti-HA antibody for 40 min, then fixed and permeabilized for immunofluorescence. Secondary antibodies used for immunofluorescence were purchased from Invitrogen. Images were acquired using a Zeiss Axioplan fluorescence microscope (Zeiss) equipped with a 63 × oil objective, a Hamamatsu Orca-R2 C10600 camera (Hamamatsu Photonics) and a SEDAT quad pass filter set (Chroma). SDS-PAGE and western blotting were performed as described by Gordon et al. (2009).

To measure the rate of VAMP internalisation, cells expressing HA-tagged VAMPs were incubated with mouse anti-HA (Covance) on ice for 30 min. The cells were washed to remove unbound antibody and warmed to 37°C for various times. Antibody uptake was halted by transferring the cells to ice. The cells were then incubated with Cy5-conjugated antibody against mouse IgG (Jackson ImmunoResearch) for 30 min. The cells were washed and the amount of bound antibody determined using an FACSCalibur flow cytometer (BD Biosciences). Live cells were separated from dead cells by 7-aminoactinomycin D exclusion. Aproximately 10,000 cells were analyzed for each sample and the mean fluorescence was calculated using the software FLOWJO (TreeStar). The percentage of uptake of bound antibody was determined by comparing cells that had been incubated at 37°C with cells that had been kept continuously on ice.

Uptake of ^{125}I-labeled transferrin and EGF was carried out as described in (Motley et al., 2003).

Protein Expression and Purification

All recombinant proteins were expressed in BL21(DE3) pLysS cells for 16 hr at 22°C after induction with 0.2 mM IPTG. Following lysis, GST fusion proteins were purified on Glutathione Sepharose 4B in 20 mM HEPES pH 7.4, 150 mM NaCl, 4 mM DTT and eluted with buffer containing 30 mM glutathione. His$_6$Myc tagged proteins were purified on Ni^{2+}-NTA agarose in 20 mM HEPES pH 7.4, 150 mM NaCl, 30 mM imidazole, 1 mM β-mercaptoethanol and eluted in the same buffer but now containing 300 mM imidazole and then subsequently supplemented with 4 mM DTT.

GST-VAMP8$_{1-76}$His$_{12}$ used for SPR was purified using 20 mM HEPES pH 7.4, 120 mM NaCl, 1 mM β-mercaptoethanol. The N-terminal GST tag was cleaved with thrombin for 3hrs at room temperature while the protein was bound to glutathione sepharose beads. The cleaved protein was then eluted and the thrombin cleavage was halted by the addition of AEBSF. The protein was further purified on Ni^{2+}-NTA agarose in 20 mM HEPES (pH 7.4), 150 mM NaCl, 30 mM imidazole, 1 mM β-mercaptoethanol and eluted in the same buffer but now containing 300 mM imidazole. Finally, it was dialysed extensively into buffer lacking both reducing agents and imidazole.

GST-CALM$_{ANTH(1-289)}$, GST-CALM$_{ANTH(1-264)}$ and GST-CALM$_{ANTH}$:VAMP8 fusion constructs destined for crystallization trials were purified using 20 mM HEPES (pH 7.4), 120 mM NaCl, 4 mM DTT. The N-terminal GST-tag was cleaved with thrombin overnight at room temperature while the protein was bound to glutathione sepharose beads. The cleaved proteins were then eluted and the thrombin cleavage was halted by the addition of AEBSF. The proteins were then further purified by S200 gel filtration and concentrated to ~15mg/ml for crystallization trials.

Recombinant Protein/Protein GST Pull-Down Experiments

Interactions were tested using appropriate GST tagged bait proteins and His_6Myc tagged prey proteins in 20 mM HEPES (pH 7.4), 150 mM NaCl, 4 mM DTT, 4 mM β-mercaptoethanol, 0.1% NP-40, 0.05% BSA. Binding experiments were carried out at 4°C overnight with constant agitation. The supernatants were removed and the beads were washed three times with 1 ml of buffer. Bound proteins were analyzed by SDS-PAGE and western blotting with anti-Myc mouse monoclonal antibody 9E10 (Santa Cruz Biotechnology) used at 1:5000, followed by Rabbit anti Mouse HRP used at 1:8000. For testing the binding to SNARE complexes, the complexes were first formed and purified in isolation before being used in binding assays. SNAP23 and syntaxin3 were themselves made by cleaving the proteins from their N-terminal GST-fusion proteins with thrombin while they were bound to glutathione sepharose beads at 25°C for 2 hr. The cleaved proteins were eluted from beads and the thrombin inactivated by the addition of ABESF. SNARE complexes containing GST-VAMP8, were made by incubating GST-VAMP8 with a three fold molar excess of SNAP23 and syntaxin3 at 4°C for 48 hr with constant agitation in 20 mM HEPES (pH 7.4), 500 mM NaCl, 4 mM DTT, 0.5% NP-40. The complex was purified on glutathione-sepharose beads and eluted with buffer containing 30 mM glutathione. Complexes were further purified on S200 gel filtration in 20 mM HEPES (pH 7.4), 150 mM NaCl, 4 mM DTT. Only fractions containing 1:1:1 complex were pooled.

Isothermal Titration Calorimetry

ITC experiments were carried out on a VPiTC machine (GE Healthcare). All proteins were purified by S200 gel filtration into 100 mM HEPES (pH 7.4), 150 mM NaCl, 4 mM DTT. SNAREs were used while still attached to GST to improve solubility. 1.4 ml of GST-SNARE (WT or mutant) was placed into the cell at 0.15 mM. $His_6MycCALM_{ANTH}$ (WT or mutant) or GST cleaved $Epsin_{ENTH}$ at 2 mM were titrated in 37 injections of 4–8 μL. The heat of dilution of $CALM_{ANTH}$ or $Epsin_{ENTH}$ into buffer was subtracted from the relevant data and the titration curves were fitted using ORIGIN software, yielding values for the stoichiometry n, equilibrium association constant K_A (= K_D^{-1}) and enthalpy of binding. All experiments were carried out at least three times and K_Ds quoted in the text are the average of all runs. Figures show a representative example of each experiment with the K_D and associated confidence of the fit (SEM) shown; n values were always within the range of 0.9 to 1.1.

Liposome-Based Binding Experiments Using Surface Plasmon Resonance

CALM binding to PtdIns4,5P_2 and VAMP8 was measured with liposomes immobilized on a L1 sensor surface of BIAcore 3000 SPR biosensor. The details of liposome generation, their immobilization on the L1 chip and the subsequent binding are described elsewhere (Jackson et al., 2010). In brief, we used liposomes composed of phosphatidylcholine (PC) and phosphatidylethanolamine (PE) at a ratio of 80% + 20% as controls. PtdIns4,5P_2 and 18:1 DGS-NTA(Ni) lipid were used at 5% at the expense of PC. Liposomes were captured on the L1 surface during 5 min injections at a flow rate of 10 μl/min. Typically the four flow cells (FCs) of the L1 chip were derivatized with following set of liposomes: PC/PE on FC1, PC/PE+ 5% PtdIns4,5P_2 on FC2, PC/PE+5% DGS-NTA(Ni) on FC3 and PC/PE+ 5% PtdIns4,5P_2+5% DGS-NTA(Ni) on FC4. To verify that liposomes on flow cells 2 and 4 contained equal amounts of PtdIns4,5P_2, the levels of $Epsin1_{ENTH}$ domain binding were monitored. The differences between the flow cells (R_{max} values) were less than 4%. After liposome capture, the surface was stabilized by two pulse injections with 50 mM NaOH for 10 s at 30 μl/min. The flow rate was set to 5 μl/min followed by injection of His-tagged VAMP8 (10 μM) for 6 min, which resulted in 600RU of captured protein on the two flow cells with the DGS-NTA(Ni) lipid. Subsequently, we monitored the binding of CALM, using one minute injections at 50 μl/min and concentrations between 10 nM and 50 μM. The kinetic parameters were calculated after background subtraction (binding to PC/PE liposomes) exactly as described (Honing et al., 2005; Jackson et al., 2010; Kelly et al., 2008). The absolute values of K_Ds obtained for binding to the individual ligands obtained by this method are around an order of magnitude lower (tighter binding) than those obtained for the same ligands in solution by ITC. This likely reflects the fact that in liposome-based SPR the ligands are effectively concentrated on a two dimensional surface rather than in solution, a situation that is more representative of the physiological situation. In this work we do not compare measurements obtained using ITC and liposome-based SPR but only compare K_D values obtained using a single technique.

Multiangle Light Scattering Experiments

Multiangle light scattering (MALS) experiments were performed immediately following size-exclusion chromatography by inline measurement of static light-scattering (DAWN HELEOS II, Wyatt Technology), differential refractive index (Optilab rEX, Wyatt Technology) and ultraviolet absorbance (Agilent 1200 UV, Agilent Techologies). Samples (100 μL) were injected onto an analytical S200 10/300 gel filtration column (GE Healthcare) equilibrated in 20 mM HEPES (pH 7.4), 120 mM NaCl, 4 mM DTT at a flow rate of 0.5 mL/min and data were analyzed using ASTRA V (Wyatt Technology).

Crystallization, Data Collection, Structure Solution, and Refinement

Crystals were grown in sitting drops containing equal amounts of protein and reservoir solution (200–400 nL) and equilibrated against 80μl of reservoir solution at 16°C as follows: $CALM_{ANTH(1-289)}$ was equilibrated against reservoirs containing 100 mM Bis Tris propane, 200 mM sodium malonate, 20% (w/v) PEG 3350; $CALM_{ANTH(1-264)}$ was equilibrated against reservoirs containing 15% v/v ethanol, 100 mM imidazole pH 8.0, 200 mM $MgCl_2$; $CALM_{ANTH(1-264)}$:$VAMP8_{(11-41)}$ was equilibrated against reservoirs containing 100 mM phosphate-citrate (pH 4.2), 200 mM NaCl, 50% (v/v) PEG-200. Crystals were cryoprotected by brief immersion (5–60 s) in reservoir solution supplemented with 25% glycerol and were rapidly cryocooled in liquid N_2 or a 100 K stream of N_2 gas.

Diffraction data were processed using MOSFLM (Leslie, 2006) and SCALA (Evans, 2006) as implemented by xia2 (Winter, 2010). Data processing statistics are summarized in Table S1. All structures were solved by molecular replacement with PHASER (McCoy et al., 2007) using previously published ANTH domain of CALM as a starting model (1HF8). Manual building was performed using COOT (Emsley and Cowtan, 2004) and structures were refined using phenix.refine (Adams et al., 2010) in consultation with the validation statistics provided by phenix.refine and Molprobity (Chen et al., 2010). Structural superpositions were performed using SSM (Krissinel and Henrick, 2004), molecular graphics were prepared using PyMOL (DeLano Scientific) and sequence alignments with ALINE (Bond and Schuttelkopf, 2009).

SUPPLEMENTAL REFERENCES

Adams, P.D., Afonine, P.V., Bunkoczi, G., Chen, V.B., Davis, I.W., Echols, N., Headd, J.J., Hung, L.W., Kapral, G.J., Grosse-Kunstleve, R.W., et al. (2010). PHENIX: a comprehensive Python-based system for macromolecular structure solution. Acta Crystallogr. D Biol. Crystallogr. *66*, 213–221.

Bond, C.S., and Schuttelkopf, A.W. (2009). ALINE: a WYSIWYG protein-sequence alignment editor for publication-quality alignments. Acta Crystallogr. D Biol. Crystallogr. *65*, 510–512.

Chen, V.B., Arendall, W.B., 3rd, Headd, J.J., Keedy, D.A., Immormino, R.M., Kapral, G.J., Murray, L.W., Richardson, J.S., and Richardson, D.C. (2010). MolProbity: all-atom structure validation for macromolecular crystallography. Acta Crystallogr. D Biol. Crystallogr. *66*, 12–21.

Diederichs, K., and Karplus, P.A. (1997). Improved R-factors for diffraction data analysis in macromolecular crystallography. Nat. Struct. Biol. *4*, 269–275.

Emsley, P., and Cowtan, K. (2004). Coot: model-building tools for molecular graphics. Acta Crystallogr. D Biol. Crystallogr. *60*, 2126–2132.

Evans, P. (2006). Scaling and assessment of data quality. Acta Crystallogr. D Biol. Crystallogr. *62*, 72–82.

Ford, M.G., Pearse, B.M., Higgins, M.K., Vallis, Y., Owen, D.J., Gibson, A., Hopkins, C.R., Evans, P.R., and McMahon, H.T. (2001). Simultaneous binding of PtdIns(4,5)P2 and clathrin by AP180 in the nucleation of clathrin lattices on membranes. Science *291*, 1051–1055.

Gordon, D.E., Mirza, M., Sahlender, D.A., Jakovleska, J., and Peden, A.A. (2009). Coiled-coil interactions are required for post-Golgi R-SNARE trafficking. EMBO Rep. *10*, 851–856.

Honing, S., Ricotta, D., Krauss, M., Spate, K., Spolaore, B., Motley, A., Robinson, M., Robinson, C., Haucke, V., and Owen, D.J. (2005). Phosphatidylinositol-(4,5)-bisphosphate regulates sorting signal recognition by the clathrin-associated adaptor complex AP2. Mol. Cell *18*, 519–531.

Jackson, L.P., Kelly, B.T., McCoy, A.J., Gaffry, T., James, L.C., Collins, B.M., Honing, S., Evans, P.R., and Owen, D.J. (2010). A large-scale conformational change couples membrane recruitment to cargo binding in the AP2 clathrin adaptor complex. Cell *141*, 1220–1229.

Kelly, B.T., McCoy, A.J., Spate, K., Miller, S.E., Evans, P.R., Honing, S., and Owen, D.J. (2008). A structural explanation for the binding of endocytic dileucine motifs by the AP2 complex. Nature *456*, 976–979.

Krissinel, E., and Henrick, K. (2004). Secondary-structure matching (SSM), a new tool for fast protein structure alignment in three dimensions. Acta Crystallogr. D Biol. Crystallogr. *60*, 2256–2268.

Leslie, A.G. (2006). The integration of macromolecular diffraction data. Acta Crystallogr. D Biol. Crystallogr. *62*, 48–57.

McCoy, A.J., Grosse-Kunstleve, R.W., Adams, P.D., Winn, M.D., Storoni, L.C., and Read, R.J. (2007). Phaser crystallographic software. J. Appl. Cryst. *40*, 658–674.

Motley, A., Bright, N.A., Seaman, M.N., and Robinson, M.S. (2003). Clathrin-mediated endocytosis in AP-2-depleted cells. J. Cell Biol. *162*, 909–918.

Weiss, M.S., Metzner, H.J., and Hilgenfeld, R. (1998). Two non-proline cis peptide bonds may be important for factor XIII function. FEBS Lett. *423*, 291–296.

Winter, G. (2010). xia2: an expert system for macromolecular crystallography data reduction. J. Appl. Cryst. *43*, 186–190.

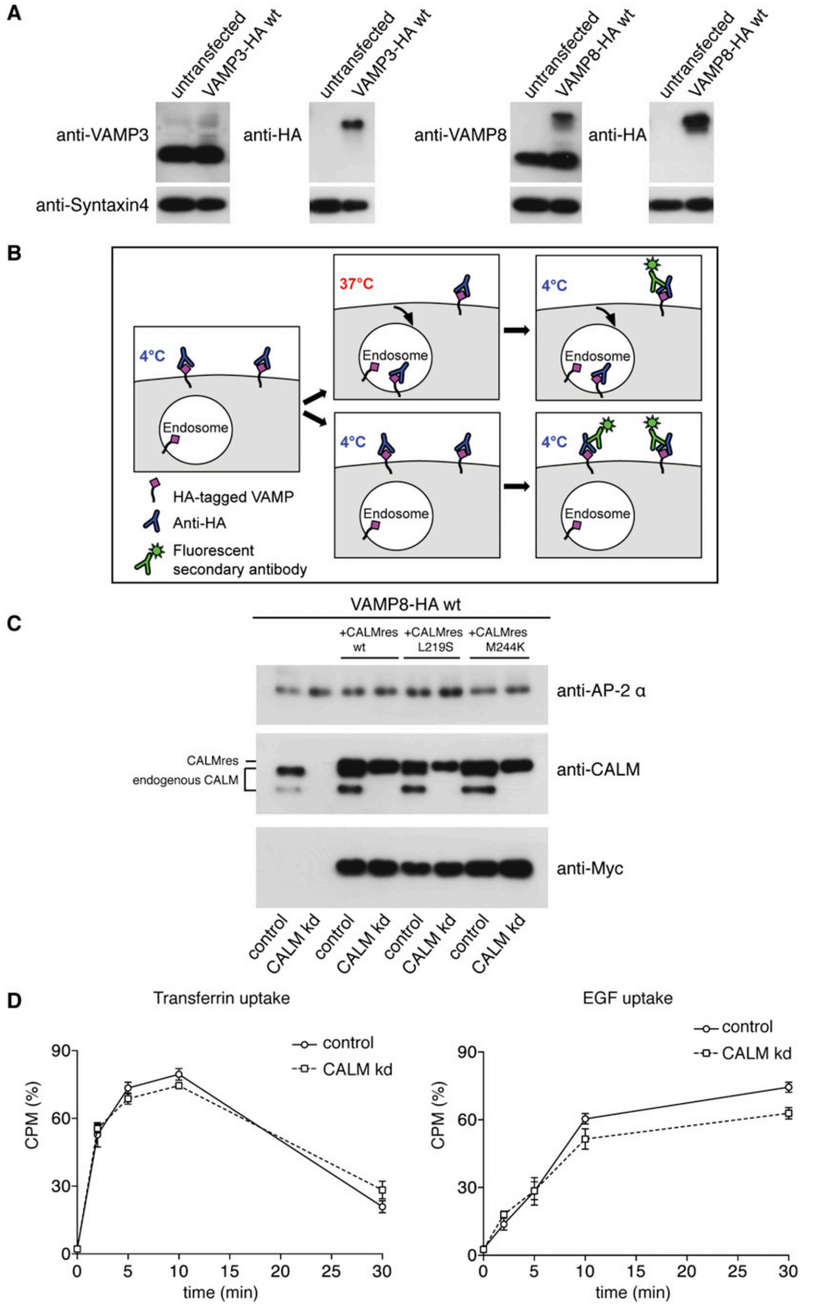

Figure S1. Endocytosis Assay, Related to Figure 1

(A) Western blot of nontransfected HeLa cells and of cells stably expressing HA-tagged VAMP3 or VAMP8, probed with an antibody against either the relevant VAMP, the HA tag, or syntaxin4 as a loading control. In both cases the tagged VAMP is expressed at considerably lower levels than the endogenous VAMP.

(B) Schematic diagram of the assay for endocytosis of HA-tagged VAMPs. Anti-HA was bound to the cell surface at 4°C, then the cells were washed and either shifted to 37°C for 2–30 min or kept at 4°C. They were then incubated with a fluorescent secondary antibody at 4°C, and the surface fluorescence was quantified by flow cytometry. A decrease in fluorescence at 37°C indicates endocytosis of the anti-HA.

(C) Western blots showing the specificity of the CALM knockdown and rescue. Extracts of cells expressing HA-tagged VAMP8 alone or coexpressing HA-tagged VAMP8 and myc-tagged siRNA-resistant CALM (wt or mutant) were probed with either anti-CALM, anti-Myc, or an antibody against the AP-2 α subunit (as a loading control). The blot shows that the three constructs are expressed at a similar level to endogenous CALM, and that the knockdown depletes endogenous CALM but not the constructs.

(D) Endocytosis of ^{125}I-labeled transferrin and EGF in control and CALM-depleted cells. The ligand was bound to the cell surface at 4°C, then the cells were shifted to 37°C for 2–30 min. The medium was harvested, ligand remaining on the plasma membrane was removed with an acid wash, the cells were solubilized with NaOH, and the label in all three was quantified. The graphs show the cell-associated counts as a percentage of the total counts. Each point is derived from at least 3 separate experiments; the error bars show the SEM.

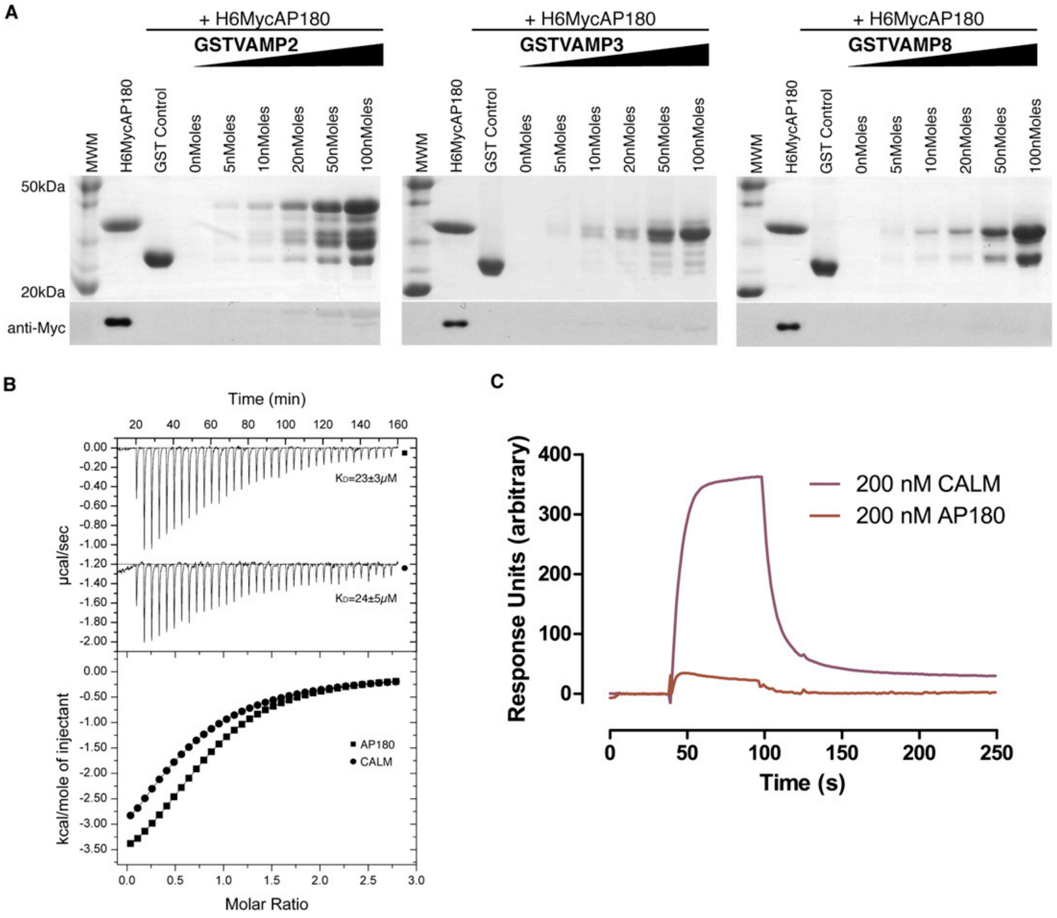

Figure S2. AP180$_{ANTH}$ Does Not Bind VAMP2, VAMP3, or VAMP8, Related to Figure 2

(A) GST Pull downs using His$_6$MycAP180$_{ANTH}$ and the GST-fusion proteins indicated. Top panel: Coomassie blue stained gel. Lower panel: western blot probed with anti-myc. The lane adjacent to the molecular weight markers (MWM) is loaded with His$_6$MycAP180$_{ANTH}$ only. The ANTH domain of AP180 does not bind to VAMP2, 3, and 8.

(B) Isothermal titration calorimetry of the binding of CALM$_{ANTH}$ and AP180$_{ANTH}$ to Ins(1,4,5)P$_3$ gave comparable binding affinities (K_D24 ± 5μM and K_D23 ± 3μM respectively) confirming both proteins were folded and functionally active. Data for CALM is translated by −1.2 μcal/s for clarity.

(C) Surface Plasmon Resonance of CALM$_{ANTH}$ and AP180$_{ANTH}$ also showed AP180$_{ANTH}$ did not bind to GST VAMP8. The SNARE was coupled directly to a CM5 chip and the binding of the ANTH domain proteins was assayed by flowing 200 nM analyte (CALM$_{ANTH}$ or AP180$_{ANTH}$) across the chip for 60 s at a flow rate of 30μl/min in 20 mM HEPES (pH 7.4), 150 mM NaCl, 4 mM DTT. Data were recorded on a Biacore 3000 (GE Healthcare) at 20°C and the binding of analyte to a mock channel (GST) has been subtracted.

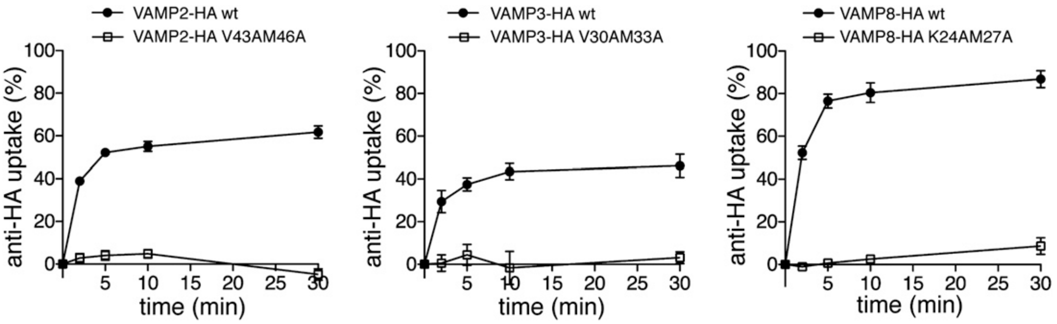

Figure S3. Analogous Mutations in the SNARE Motifs of VAMP2, VAMP3, and VAMP8 Abolish Their Endocytosis, Related to Figure 3

Endocytosis of anti-HA in cells expressing HA-tagged VAMPs, using the flow cytometry assay described in Figure S1B. The wild-type constructs were efficiently internalized, but the VAMP2-HAV43AM46A mutant, the VAMP3-HAV30AM33A mutant, and the VAMP8-HAK24AM27A mutant remained on the plasma membrane.

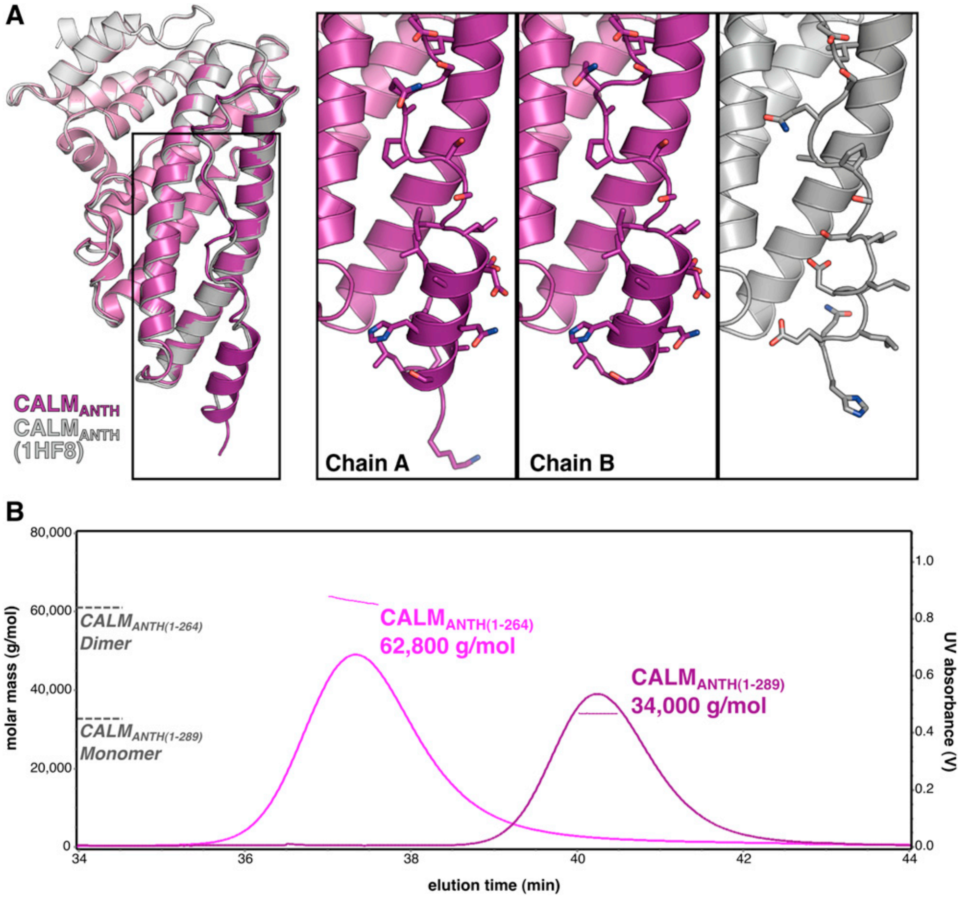

Figure S4. Superposition of $CALM_{ANTH}$ Domains and Dimerization of $CALM_{ANTH(1\text{-}264)}$, Related to Figure 4

(A) The structure of $CALM_{ANTH(1\text{-}289)}$ (purple) from this study overlays with the previously published structure of $CALM_{ANTH}$ (1HF8; gray) (Ford et al., 2001) with Cα atom rmsds of 0.43 Å (chain A) and 0.47 Å (chain B). Insets highlight the poorly-ordered C-terminal section for the structures of the ANTH domains.

(B) The weight-averaged molar mass (thin lines) is shown across the elution profile ($A_{280\ nm}$, thick lines) of $CALM_{ANTH}$ constructs subjected to size-exclusion chromatography and MALS. The expected molar masses of $CALM_{ANTH(1\text{-}289)}$ monomers (M_r = 33,486) and $CALM_{ANTH(1\text{-}264)}$ dimers (M_r = 62,792) are shown for reference (dotted grey lines).

Cell

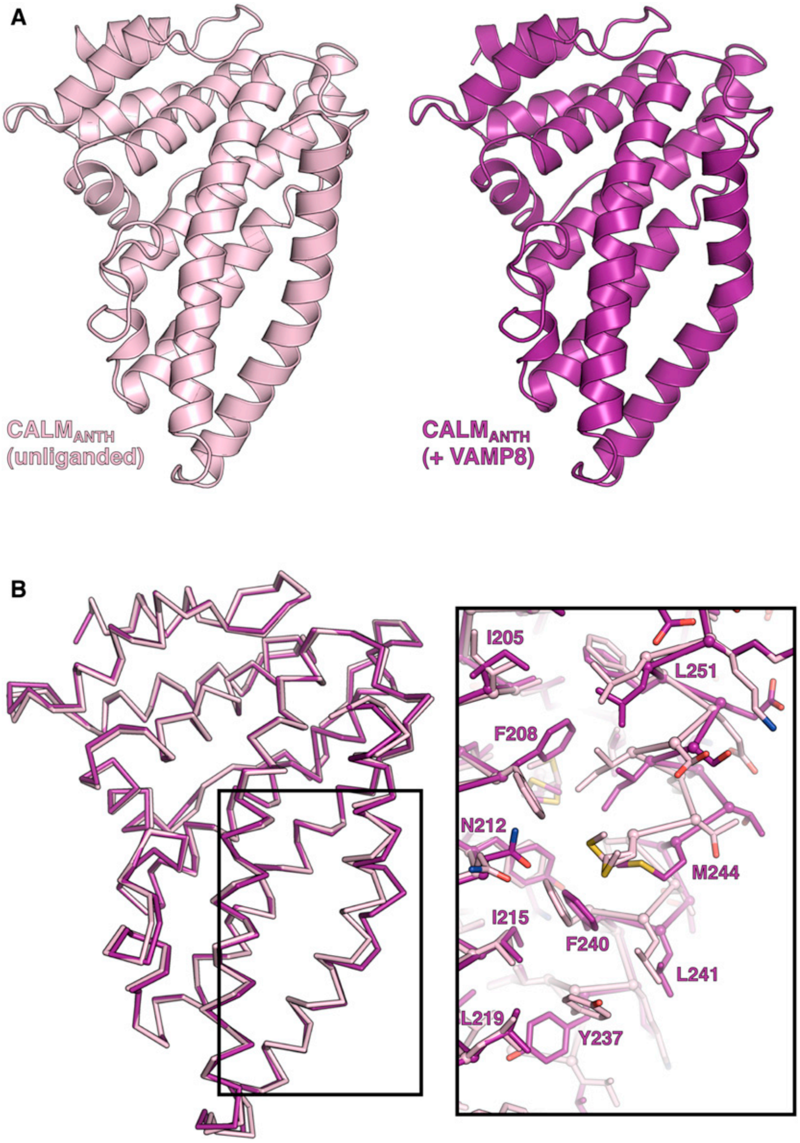

Figure S5. CALM$_{ANTH}$ Does Not Undergo Major Structural Reorganization upon Binding VAMP8, Related to Figure 5
(A) Residues 19–264 of the ANTH domains of unbound CALM (pink) and VAMP8-bound CALM (purple) shown in ribbon representation.
(B) Superimposition of Cα traces of residues 19–264 of unbound CALM (pink) and VAMP8-bound (purple) CALM$_{ANTH}$. Inset shows reorganisation of side chains upon binding at the VAMP8 interaction interface colored as above.

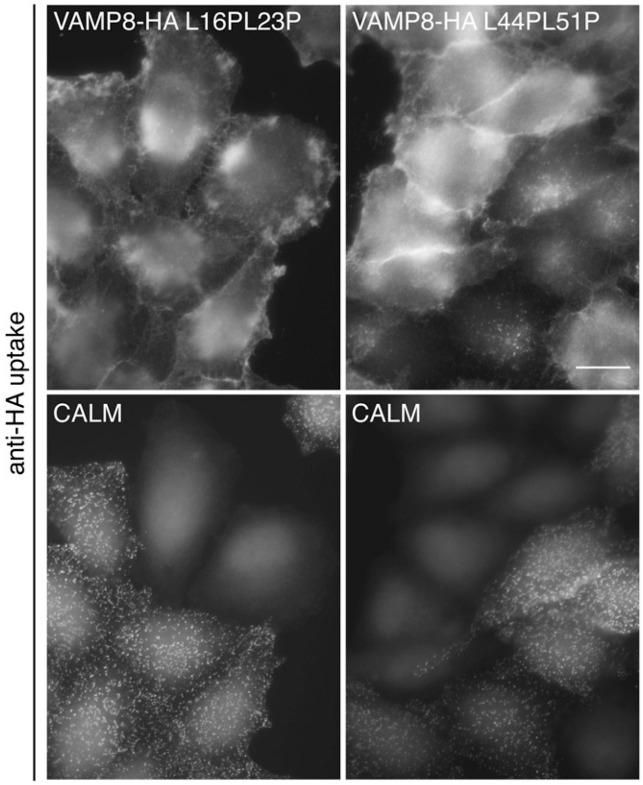

Figure S6. Localization of Anti-HA in Cells Expressing Different VAMP8-HA Proline Mutants, Related to Figure 7

Control and siRNA-treated cells were mixed together and allowed to endocytose the antibody for 40 min, then processed for immunofluorescence. Knocking down CALM prevents antibody uptake in cells expressing the L44PL51P mutant, but in cells expressing the L16PL23P mutant the antibody stays on the cell surface whether or not CALM is knocked down. The scale bar represents 20 μm.
